# Stratified survival of resected and overall pancreatic cancer patients in Europe and the USA in the early twenty-first century: a large, international population-based study

**DOI:** 10.1186/s12916-018-1120-9

**Published:** 2018-08-21

**Authors:** Lei Huang, Lina Jansen, Yesilda Balavarca, Masoud Babaei, Lydia van der Geest, Valery Lemmens, Liesbet Van Eycken, Harlinde De Schutter, Tom B. Johannesen, Maja Primic-Žakelj, Vesna Zadnik, Marc G. Besselink, Petra Schrotz-King, Hermann Brenner

**Affiliations:** 1 Division of Clinical Epidemiology and Aging Research, German Cancer Research Center (DKFZ), Im Neuenheimer Feld 581, 69120 Heidelberg, Germany; 2 Medical Faculty Heidelberg, Heidelberg University, Heidelberg, Germany; 3 German Cancer Consortium (DKTK), German Cancer Research Center (DKFZ), Heidelberg, Germany; 4 Division of Preventive Oncology, DKFZ and National Center for Tumor Diseases (NCT), Heidelberg, Germany; 5 The Netherlands Cancer Registry, The Netherlands Comprehensive Cancer Organization (IKNL), Utrecht, the Netherlands; 6 Belgian Cancer Registry (BCR), Brussels, Belgium; 7 Cancer Registry of Norway (CRN), Oslo, Norway; 8 Cancer Registry of Slovenia (CRS), Ljubljana, Slovenia; 9 Dutch Pancreatic Cancer Group, Amsterdam UMC, University of Amsterdam, Amsterdam, the Netherlands

**Keywords:** Pancreatic cancer, Resection, Survival, TNM stage, Age, Population-based, Early twenty-first century

## Abstract

**Background:**

The prognosis of pancreatic cancer (PaC) strongly varies across different stages and age groups, which has unfortunately not been well recorded in the literature. This international population-based study aimed to provide tumor-node-metastasis (TNM) stage- and age-specific survival estimates and trends in resected and overall (resected and unresected) PaC in the early twenty-first century.

**Methods:**

Using data from the US Surveillance, Epidemiology, and End Results-18 Program and the national cancer registries of the Netherlands, Belgium, Norway, and Slovenia, short-term and long-term overall survival results stratified by TNM stage and age in resected and overall primary PaC, *irrespective of being microscopically confirmed or not*, in 2003–2014 were computed using the Kaplan-Meier method. The temporal survival trends over three predefined periods (2003–2005, 2006–2008, and 2009–2011) were further examined using the log-rank test.

**Results:**

In total, data for 125,183 patients were analyzed. Overall, age-stratified 3-year survival was 20–34% (< 60 years), 14–25% (60–69 years), and 9–13% (≥ 70 years) in stages I–II PaC; and 2–5% (< 60 years), 1–2% (60–69 years), and < 1–1% (≥ 70 years) in stages III–IV cancer. Patients who underwent operation had higher 3-year survival in each stage and age group (stages I–II: 23–39% (< 60 years), 16–31% (60–69 years), and 17–30% (≥ 70 years); stages III–IV: 5–19% (< 70 years) and 2–14% (≥ 70 years)). Perioperative survival also decreased with advancing stage and older age (stages I–II: 98–100% (< 60 years), 97–99% (60–69 years), and 94–99% (≥ 70 years); stages III–IV: 94–99% (< 70 years) and 81–96% (≥ 70 years)). Between 2003 and 2005 and 2009–2011, for overall PaC, both short-term and long-term survival improvements were observed in all countries except Belgium; for resected disease, short-term improvements were present only in the USA and Slovenia, but long-term improvements were observed in all countries except Slovenia, with stage-specific variations.

**Conclusions:**

Our large international study provides TNM stage- and age-specific population-based survival in overall and resected PaC that will facilitate clinical counseling. While the survival expectations for patients with resected PaC are substantially higher than the widely available and known dismal survival predictions for overall patients, conclusions on the benefits of resection cannot be made from this observational study. Patients with advanced-stage disease and/or older age should undergo careful risk assessment before treatment. Limited but inspiring improvement in survival is observed.

**Electronic supplementary material:**

The online version of this article (10.1186/s12916-018-1120-9) contains supplementary material, which is available to authorized users.

## Background

Pancreatic cancer (PaC) is one of the deadliest malignancies worldwide; > 340,000 individuals receive this diagnosis annually with similar incidence and mortality rates [1]. PaC incidence is especially high in developed countries, being the fourth leading cause of cancer-related deaths in Western societies [2]. In the EU, PaC incidence has been stable or moderately increasing over the past decades, and it was estimated to have caused 91,500 deaths in 2017 and to cause 111,500 deaths in 2025, potentially becoming the third leading cause of cancer death [3].

The prognosis for PaC is poor, with 5-year survival of only ~ 5% [4]. Due to lack of effective early screening methods, more than half of PaCs are detected at advanced stages and are treated largely palliatively [5, 6]. According to the current guidelines [7–11], only tumor-node-metastasis (TNM) stages I–II PaCs are usually resectable, although the resectability criteria are differentially and arguably defined [12]. While resection could markedly improve the long-term survival in selected patients, less than one-fifth of diagnosed cases are considered eligible for resection [5, 10]. Patients with resectable PaC who undergo resection have much better survival rates than those who cannot undergo resection [8, 10].

We previously described the low and varying resection rates for PaC in Europe and the USA [13]. When counseling PaC patients who are considering surgery or who have already undergone resection, it is important to provide survival estimates for the resected subgroup. However, at this time, population-based survival estimates are only available for overall patients without differentiation by resection or TNM stage [4], according to which, however, survival might vary greatly. Survival odds in resected PaC data from institutional reports would be accompanied with relatively high patient selection, making the generalizability questionable. Based on multiple national databases, this large investigation aimed to comprehensively and robustly provide 1-month to 5-year overall survival estimates at the population level for overall (resected and unresected) and resected PaC patients diagnosed in the early twenty-first century in Europe and the USA stratified by TNM stage and age. Furthermore, survival trends over time in each country were explored.

## Methods

### Study design

A list of all contacted cancer registries together with reasons for exclusion are provided in Additional file 1: Table S1. Population-based PaC data from the US Surveillance, Epidemiology, and End Results (SEER)-18 Program, the Netherlands Cancer Registry, the Belgian Cancer Registry, the Cancer Registry of Norway, and the Cancer Registry of Slovenia were investigated in this large real-world observational study (Additional file 1: Table S2). The data quality of each registry was described in a previous publication [13], focusing on resection rates only. Among the previous participants [13], the Danish registry withdrew its participation due to some legislation issues, and the small population size for resected patients in Estonia did not allow for reasonable survival analyses. Institution-based data were not included due to the relatively high risk of patient selection bias. The participating European national registries, located in western, northern, and southern Europe, respectively, were those able to provide data of relatively high quality, according to a uniform data-request sheet, to ensure the robustness of the results. All variables were uniformly (re)coded across registries. National population-based registries were not included if they were not able to provide eligible treatment, TNM staging, or survival data. This study was approved by the Ethics Committee of Medical Faculty Heidelberg and reported following the Strengthening the Reporting of Observational Studies in Epidemiology (STROBE) guidelines.

### Patients

Only patients with pathologic and/or clinical diagnoses of invasive primary malignant tumors of the exocrine pancreas were included. Patients were included irrespective of being microscopically diagnosed or not in this real-world study on survival for resected and overall PaC following the EUROCARE studies [4, 14], since consensus has been reached by the International Study Group of Pancreatic Surgery (ISGPS) that, in the presence of a solid mass suspicious for malignancy, biopsy proof has not been and is not required before proceeding with resection [15]. Those with benign/premalignant tumors, non-pancreatic neoplasms involving the pancreas, neuroendocrine tumors, carcinoids, sarcomas/stromal tumors, germ cell neoplasms, lymphomas, or periampullar tumors (Additional file 1: Table S3), with diagnosis based on autopsy or death certificate only, or with unknown diagnosis/follow-up date or survival status were excluded. Patients without TNM staging were also excluded. As the fifth edition of the TNM staging system was incompatible with the later versions in effect during 2003–2017 [8], only patients with PaC diagnosed after 2002 were included.

### Collected information

Information on patient (year of diagnosis, sex, and age) and tumor characteristics (microscopic confirmation, TNM stage, location, and differentiation), treatment (resection, (neo) adjuvant chemotherapy, and radiotherapy), follow-up, and survival status was obtained. Tumor morphology and topography were coded according to the International Classification of Diseases for Oncology, Third Edition. Tumor stage was based on the TNM staging system, sixth/seventh edition [8]. In stage classification, pathologic (pTNM) stages were prioritized over clinical (cTNM) ones. Resection was defined as surgical removal of the primary tumor, regardless of being curative or palliative. Survival status was obtained from official population registers and/or national death registrations.

### Outcome measures

Short-term (1-month to 6-month) and relatively long-term (1-year to 5-year) survival data in overall and resected PaC stratified by TNM stage (I–II and III–IV) and age group (< 60, 60–69, and ≥ 70 years) were presented. Cancer stage was divided into stages I–II and III–IV, considering the former to be clearly resectable and the latter mostly unresectable, and to ensure an adequate number for assessment in each subgroup. When describing survival for resected stages III–IV PaC, the subgroups < 60 and 60–69 years were combined considering the small size of either. Survival trends over 3 calendar years (2003–2005, 2006–2008, and 2009–2011) in each country were further reported. All these categories were predefined.

### Statistical analyses

Complete-case analysis was performed for patients with known TNM stages. Results were described for each country separately without pooling, considering the potential heterogeneity across countries. Overall survival was defined as the months between diagnosis and death from any cause/last follow-up, and was estimated for overall and resected PaC patients by TNM stage and age group using the Kaplan-Meier method, with the 1-, 3-, 6-, 12-, 24-, 36-, and 60-month survival rates calculated. Sensitivity analyses were conducted by limiting the overall patients to those with microscopic confirmation. Changes in survival rates of overall and operated patients diagnosed between 2003 and 2005 and 2009–2011 were examined using the log-rank test. Statistical significance was defined by two-sided *P* <  0.05. The SAS software (version 9.4, Cary, NC, USA) was used.

## Results

### Patient characteristics

In total, data for 125,183 patients (stages I–II, 42,955 (34%); stages III–IV, 82,228 (66%)) were analyzed. Patients were diagnosed in comparable periods in all countries (2003/2004 until 2013/2014). Demographic and clinical characteristics for overall and operated patients with stages I–II and III–IV PaCs are shown in Table 1 and described in Additional file 1. In overall PaCs, 66% (Norway) to 91% (Belgium) of stages I–II cancers and 53% (Slovenia) to 86% (Belgium) of stages III–IV cancers were microscopically confirmed. Nearly all resected PaCs were microscopically confirmed (stages I–II, 99– ≥ 99%; stages III–IV, 92–100%).

**Table 1:** Demographic and clinical characteristics of patients with stages I–II and III–IV pancreatic cancer

Parameter	USA (2004–2013)		The Netherlands (2003–2014)		Belgium (2004–2013)		Norway (2003–2014)		Slovenia (2003–2013)	
Group	Overall	Resected	Overall	Resected	Overall	Resected	Overall	Resected	Overall	Resected
Stages I–II										
Number (n)	31,313	13,303 (43)	5710	2675 (47)	3437	2155 (63)	1545	526 (34)	667	406 (61)
Microscopically confirmed	27,290 (87)	13,290 (> 99)	4046 (71)	2673 (> 99)	3127 (91)	2148 (> 99)	1017 (66)	520 (99)	475 (71)	401 (99)
Gender, female	16,193 (52)	6604 (50)	2951 (52)	1268 (47)	1684 (49)	993 (46)	853 (55)	261 (50)	361 (54)	209 (52)
Age, years	70 ± 12	66 ± 11	71 ± 11	65 ± 10	69 ± 11	66 ± 10	72 ± 12	65 ± 11	69 ± 11	65 ± 10
Age group										
< 60 years	6100 (20)	3574 (27)	981 (17)	693 (26)	686 (20)	546 (25)	234 (15)	134 (26)	144 (22)	122 (30)
60–69 years	7817 (25)	4272 (32)	1477 (26)	978 (37)	937 (27)	698 (32)	375 (24)	195 (37)	166 (25)	131 (32)
≥ 70 years	17,396 (56)	5457 (41)	3252 (57)	1004 (38)	1814 (53)	911 (42)	936 (61)	197 (37)	357 (54)	153 (38)
Tumor location^a^										
Pancreas head	22,412 (83)	9573 (80)	4666 (89)	2187 (89)	1807 (81)	1207 (82)	851 (85)	394 (84)	471 (88)	321 (87)
Pancreas body	2502 (9)	890 (8)	255 (5)	91 (4)	179 (8)	100 (7)	87 (9)	33 (7)	35 (7)	26 (7)
Pancreas tail	2196 (8)	1448 (12)	296 (6)	191 (8)	244 (11)	169 (11)	62 (6)	41 (9)	32 (6)	21 (6)
Other	4203 (13)	1392 (11)	493 (9)	206 (8)	1207 (35)	679 (32)	545 (35)	58 (11)	129 (19)	38 (9)
Differentiation^b^										
Well	2145 (13)	1428 (12)	283 (12)	244 (11)	389 (18)	302 (17)	61 (10)	27 (6)	41 (10)	37 (10)
Intermediate	7574 (47)	6026 (51)	1225 (51)	1138 (52)	1072 (48)	901 (50)	352 (55)	259 (61)	153 (37)	142 (38)
Poor/undifferentiated	6264 (39)	4464 (38)	920 (38)	802 (37)	762 (34)	608 (34)	223 (35)	135 (32)	221 (53)	192 (52)
Neoadjuvant chemotherapy	–	NA	–	50 (2)	–	53 (3)	–	NA	–	2 (1)
Neoadjuvant radiotherapy	–	522 (4)	–	34 (1)	–	20 (1)	–	0 (0)	–	1 (< 1)
Resection type										
Pancreatoduodenectomy	–	9479 (71)	–	2269 (85)	–	NA	–	NA	–	NA
Distal pancreatectomy	–	1878 (14)	–	256 (10)	–	NA	–	NA	–	NA
Total pancreatectomy	–	1629 (12)	–	42 (2)	–	NA	–	NA	–	NA
Other^c^	–	314 (2)	–	108 (4)	–	NA	–	NA	–	NA
Adjuvant/palliative chemotherapy	NA	NA	1392 (24)	1078 (40)	1796 (52)	1200 (56)	265 (17)	127 (24)	131 (20)	120 (30)
Adjuvant/palliative radiotherapy	4460 (14)	4193 (32)	120 (2)	33 (1)	326 (10)	190 (9)	64 (4)	17 (3)	9 (1)	8 (2)
Stages III–IV										
Number (n)	55,153	1998 (4)	13,974	237 (2)	5632	298 (5)	4633	108 (2)	1997	135 (7)
Microscopically confirmed	46,973 (85)	1994 (> 99)	10,375 (74)	237 (100)	4837 (86)	297 (> 99)	3224 (70)	105 (97)	1056 (53)	124 (92)
Gender, female	26,427 (48)	969 (49)	6755 (48)	106 (45)	2700 (48)	154 (52)	2332 (50)	47 (44)	967 (48)	56 (42)
Age, years	69 ± 12	65 ± 12	68 ± 11	64 ± 10	69 ± 11	64 ± 10	71 ± 12	64 ± 10	69 ± 11	65 ± 10
Age group										
< 60 years	12,582 (23)	636 (32)	2938 (21)	69 (29)	1043 (19)	92 (31)	759 (16)	32 (30)	393 (20)	40 (30)
60–69 years	15,081 (27)	622 (31)	4412 (32)	96 (41)	1608 (29)	103 (35)	1268 (27)	43 (40)	513 (26)	45 (33)
≥ 70 years	27,490 (50)	740 (37)	6624 (47)	72 (30)	2981 (53)	103 (35)	2606 (56)	26 (24)	1091 (55)	43 (32)
Tumor location^a^										
Pancreas head	21,244 (56)	1069 (66)	7202 (62)	166 (79)	1585 (58)	126 (69)	1245 (63)	57 (66)	653 (64)	80 (78)
Pancreas body	7893 (21)	168 (10)	1844 (16)	11 (5)	508 (19)	19 (10)	340 (17)	8 (9)	160 (16)	12 (12)
Pancreas tail	9003 (24)	381 (24)	2666 (23)	34 (16)	649 (24)	37 (20)	382 (19)	21 (24)	215 (21)	10 (10)
Other	17,013 (31)	380 (19)	2262 (16)	26 (11)	2890 (51)	116 (39)	2666 (58)	22 (20)	969 (49)	33 (24)
Differentiation^d^										
Well	1213 (10)	173 (11)	222 (9)	20 (11)	425 (17)	42 (17)	122 (8)	6 (7)	33 (8)	6 (7)
Intermediate	4518 (35)	680 (45)	833 (35)	98 (51)	962 (39)	113 (47)	596 (40)	50 (61)	112 (25)	30 (34)
Poor/undifferentiated	6795 (56)	670 (44)	1340 (56)	73 (38)	1082 (44)	88 (36)	765 (52)	26 (32)	297 (67)	52 (59)
Neoadjuvant chemotherapy	–	NA	–	15 (6)	–	24 (8)	–	NA	–	0 (0)
Neoadjuvant radiotherapy	–	139 (7)	–	5 (2)	–	9 (3)	–	0 (0)	–	0 (0)
Resection type										
Pancreatoduodenectomy	–	1140 (57)	–	178 (75)	–	NA	–	NA	–	NA
Distal pancreatectomy	–	295 (15)	–	39 (17)	–	NA	–	NA	–	NA
Total pancreatectomy	–	211 (11)	–	5 (2)	–	NA	–	NA	–	NA
Other^c^	–	352 (18)	–	15 (6)	–	NA	–	NA	–	NA
Adjuvant/palliative chemotherapy	NA	NA	3475 (25)	81 (34)	3661 (65)	195 (65)	1159 (25)	25 (23)	368 (18)	39 (29)
Adjuvant/palliative radiotherapy	770 (1)	383 (19)	328 (2)	7 (3)	358 (6)	35 (12)	198 (4)	4 (4)	46 (2)	5 (4)

*NOS* not otherwise specified, *NA* not available; −, not applicable

Enumeration data are shown as count (percentage [%]), and measurement data as mean ± standard deviation. Records are complete, otherwise specified below

^a^The percentages of pancreas head, body, and tail are the proportions compared to the total tumor cases of the 3 locations; ”other” includes pancreas duct, overlapping lesion, NOS, and other specified parts, and its proportion is relative to the whole cases

^b^Unknown differentiation in stages I–II cancer: USA, overall 15,330 (49%), resected 1385 (10%); the Netherlands, overall 3282 (58%), resected 491 (18%); Belgium, overall 1214 (35%), resected 344 (16%); Norway, overall 909 (59%), resected 100 (19%); Slovenia, overall 252 (38%), resected 35 (9%); Estonia, overall 134 (47%), resected 32 (20%)

^c^Pancreatectomy (NOS) and local resection

^d^Unknown differentiation in stages III–IV cancer: USA, overall 42,627 (77%), resected 475 (24%); the Netherlands, overall 11,579 (83%), resected 46 (19%); Belgium, overall 3163 (56%), resected 55 (19%); Norway, overall 3150 (68%), resected 26 (24%); Slovenia, overall 1555 (78%), resected 47 (35%); Estonia, overall 718 (86%), resected 5 (28%)

### Survival in overall and resected stages I–II PaCs

**Fig. 1:**
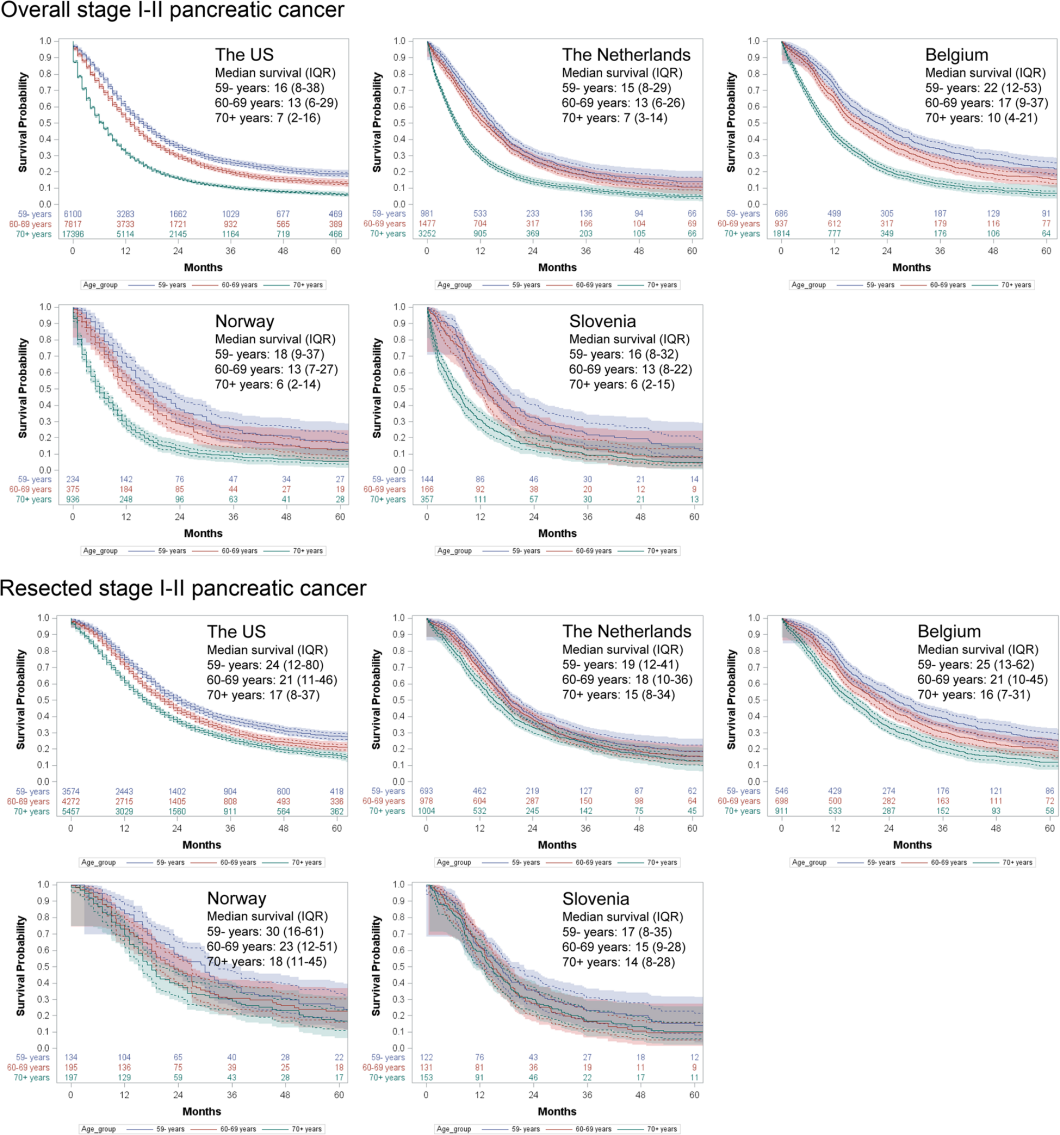
Kaplan-Meier curves (*solid lines*) of age group-specific survival in overall (*upper panel*) and resected patients (*lower panel*) with TNM stages I–II pancreatic cancers. The *dashed lines* indicate the 95% confidence limits, and the *shadows* represent the Hall-Wellner confidence bands. Numbers of patients at risk are also reported. Median survival is in months. *IQR* interquartile range

Survival in overall and resected stages I–II tumors is shown in Fig. 1, and the corresponding 1-month to 5-year survival rates are detailed in Table 2. For total patients, survival was lower in older patients and decreased strongly after diagnosis, with 3-year rates of 20–34% (< 60 years), 14–25% (60–69 years), and 9–13% (≥ 70 years), respectively. The subgroup of resected patients of all age groups in all countries had higher survival estimates, with 1-month (perioperative) rates of 98–100% (< 60 years), 97–99% (60–69 years), and 94–99% (≥ 70 years); and 3-year rates of 23–39% (< 60 years), 16–31% (60–69 years), and 17–30% (≥ 70 years), respectively. Again, younger patients had a better prognosis than older ones. However, age-specific differences were smaller, especially between those aged 60–69 and ≥ 70 years.

**Table 2:** Unadjusted survival proportions in patients with overall and resected stages I–II pancreatic cancer

Time	USA		The Netherlands		Belgium		Norway		Slovenia	
	Overall	Resected	Overall	Resected	Overall	Resected	Overall	Resected	Overall	Resected
	OS (95% CI)^a^	OS (95% CI)	OS (95% CI)	OS (95% CI)	OS (95% CI)	OS (95% CI)	OS (95% CI)	OS (95% CI)	OS (95% CI)	OS (95% CI)
1 month										
< 60 years	95 (94–95)	98 (98–99)	97 (96–98)	98 (97–99)	98 (97–99)	99 (97–99)	98 (96–99)	100 (100–100)	97 (93–99)	98 (93–99)
60–69 years	92 (91–93)	97 (96–97)	95 (94–96)	97 (96–98)	97 (96–98)	98 (97–99)	97 (94–98)	99 (95–100)	96 (92–98)	99 (94–100)
≥ 70 years	79 (78–79)	94 (93–94)	88 (87–89)	96 (95–97)	93 (92–94)	96 (94–97)	89 (86–90)	99 (95–100)	85 (81–89)	99 (95–100)
3 months										
< 60 years	89 (88–90)	96 (95–97)	92 (90–94)	97 (95–98)	95 (93–96)	96 (94–98)	95 (92–97)	99 (95–100)	92 (86–95)	94 (88–97)
60–69 years	84 (84–85)	94 (93–95)	87 (85–89)	94 (93–96)	93 (91–94)	94 (92–96)	90 (86–93)	97 (93–99)	90 (84–94)	96 (91–98)
≥ 70 years	65 (64–66)	89 (88–90)	72 (70–73)	91 (89–92)	83 (81–84)	90 (88–92)	68 (65–71)	95 (91–98)	66 (61–71)	88 (82–92)
6 months										
< 60 years	80 (79–81)	91 (90–92)	81 (78–83)	92 (90–94)	90 (87–92)	93 (90–95)	86 (81–90)	96 (90–98)	88 (81–92)	89 (82–94)
60–69 years	73 (72–74)	88 (86–89)	76 (74–78)	89 (86–91)	86 (83–88)	89 (87–91)	77 (72–81)	91 (86–95)	78 (71–84)	85 (77–90)
≥ 70 years	51 (50–51)	80 (79–81)	53 (51–55)	81 (78–83)	67 (65–69)	80 (77–83)	50 (47–53)	88 (83–92)	50 (45–55)	82 (75–88)
12 months										
< 60 years	59 (57–60)	75 (73–76)	60 (56–63)	74 (70–77)	73 (70–76)	78 (75–82)	64 (57–70)	82 (75–88)	60 (52–68)	63 (54–71)
60–69 years	52 (51–53)	71 (69–72)	53 (50–55)	68 (65–71)	65 (62–68)	72 (68–75)	52 (47–57)	73 (66–79)	55 (48–63)	62 (53–70)
≥ 70 years	31 (31–32)	61 (59–62)	30 (29–32)	61 (57–64)	43 (40–45)	58 (55–62)	28 (25–31)	70 (63–76)	31 (26–36)	60 (51–67)
24 months										
< 60 years	35 (34–37)	50 (48–52)	30 (27–33)	40 (36–44)	47 (43–51)	53 (49–57)	38 (32–45)	58 (48–66)	33 (25–41)	36 (27–44)
60–69 years	29 (28–30)	44 (42–45)	28 (25–30)	38 (35–42)	38 (35–41)	45 (41–48)	29 (24–34)	48 (40–55)	23 (17–30)	28 (20–35)
≥ 70 years	16 (15–16)	37 (35–38)	14 (13–15)	33 (30–37)	21 (19–23)	33 (30–36)	13 (10–15)	38 (31–45)	16 (12–20)	30 (23–37)
36 months										
< 60 years	26 (24–27)	37 (36–39)	20 (18–23)	27 (24–31)	34 (30–37)	39 (35–43)	26 (20–32)	39 (30–48)	22 (16–29)	23 (16–31)
60–69 years	20 (19–21)	31 (29–32)	18 (16–20)	25 (22–28)	25 (22–28)	30 (27–34)	18 (14–23)	31 (24–38)	14 (9–19)	16 (11–23)
≥ 70 years	10 (10–11)	26 (24–27)	9 (8–11)	24 (21–27)	13 (11–14)	21 (19–24)	9 (7–11)	30 (23–37)	9 (7–13)	17 (11–23)
60 months										
< 60 years	19 (18–20)	28 (26–30)	14 (12–17)	19 (16–23)	23 (19–26)	26 (22–30)	17 (12–23)	25 (17–34)	15 (9–21)	15 (9–23)
60–69 years	13 (12–14)	21 (20–23)	11 (9–13)	16 (13–19)	16 (14–19)	20 (17–23)	13 (9–17)	23 (16–30)	8 (5–13)	10 (5–16)
≥ 70 years	6 (6–7)	16 (15–17)	5 (4–6)	13 (10–16)	7 (6–8)	12 (10–15)	6 (4–7)	17 (11–24)	5 (3–8)	10 (6–16)

*OS* overall survival, *CI* confidence interval

^a^Data are shown as survival proportion (95% confidence interval) [%]

### Survival in overall and resected stages III–IV PaCs

Considering the potentially varying proportions of underreporting of advanced-stage disease, survival results for stages III–IV PaCs should be interpreted with caution. Survival in overall and resected stages III–IV cancers is shown in Additional file 1: Figure S1, and the corresponding 1-month to 5-year survival rates are detailed in Additional file 1: Table S4. Generally, patients with stages III–IV PaCs had much lower survival results than those with stages I–II tumors, and already had high mortality shortly after diagnosis. In the total group, survival decreased with increasing age, with 3-year rates of 2–5% (< 60 years), 1–2% (60–69 years), and 1–1% (≥ 70 years), respectively. The resected subgroups showed higher survival estimates than the overall population in all countries and all age groups (perioperative rates: < 70 years, 94–99%; ≥ 70 years, 81–96%; 3-year rates: < 70 years, 5–19%; ≥ 70 years, 2–14%). Differences between age groups were smaller in the resected subgroups than the overall patient population.

### Survival in overall stages I–II and III–IV PaCs with microscopic confirmation

Considering the relatively high proportions of non-microscopically confirmed overall PaC patients, we conducted sensitivity analyses by limiting the patients with overall stages I–II and III–IV cancer to those with microscopic confirmation (Table 3 and Additional file 1: Figure S2). Microscopically confirmed patients with stages I–II and III–IV cancer generally had higher survival especially in those ≥ 70 years old and within 24 months after diagnosis, in all participating countries except Belgium, where microscopic confirmation rates were high and where survival remained very similar. The 3-year survival rates remained mostly similar to those of the main analyses, and were 21–34% (< 60 years), 14–25% (60–69 years), and 12–14% (≥ 70 years) in stages I–II PaC, and 2–5% (< 60 years), 1–2% (60–69 years), and 1%–1% (≥ 70 years) in stages III–IV cancer.

**Table 3:** Unadjusted survival proportions in overall microscopically confirmed stages I–II and III–IV pancreatic cancer patients

Time	USA		The Netherlands		Belgium		Norway		Slovenia	
	Stages I–II	Stages III–IV	Stages I–II	Stages III–IV	Stages I–II	Stages III–IV	Stages I–II	Stages III–IV	Stages I–II	Stages III–IV
	OS (95% CI)^a^	OS (95% CI)	OS (95% CI)	OS (95% CI)	OS (95% CI)	OS (95% CI)	OS (95% CI)	OS (95% CI)	OS (95% CI)	OS (95% CI)
1 month										
≤ 60 years	96 (95–96)	77 (76–78)	98 (97–99)	87 (86–89)	98 (97–99)	93 (91–95)	99 (96–100)	92 (90–94)	98 (93–99)	87 (82–90)
60–69 years	93 (92–93)	72 (71–72)	96 (95–97)	83 (82–84)	97 (96–98)	89 (87–91)	97 (94–98)	87 (85–89)	97 (92–99)	82 (77–86)
≥ 70 years	84 (84–85)	60 (59–61)	92 (90–93)	74 (72–75)	93 (92–94)	81 (80–83)	96 (94–98)	80 (78–82)	94 (90–97)	67 (63–72)
3 months										
≤ 60 years	90 (90–91)	59 (58–60)	93 (91–94)	61 (59–63)	95 (93–96)	78 (75–80)	96 (93–98)	67 (63–70)	95 (90–98)	61 (55–67)
60–69 years	86 (85–87)	53 (53–54)	89 (87–91)	55 (53–57)	92 (91–94)	69 (67–71)	91 (88–94)	59 (56–62)	93 (88–96)	54 (48–59)
≥ 70 years	71 (71–72)	40 (39–40)	79 (77–81)	42 (41–44)	83 (81–84)	55 (53–56)	85 (82–88)	44 (42–47)	81 (75–86)	39 (35–44)
6 months										
≤ 60 years	81 (80–82)	41 (40–42)	83 (81–86)	36 (34–37)	90 (87–92)	59 (56–62)	88 (82–91)	43 (39–46)	92 (85–95)	44 (38–49)
60–69 years	74 (73–75)	35 (35–36)	79 (77–81)	32 (31–34)	86 (83–88)	50 (48–53)	79 (74–83)	37 (34–40)	82 (74–87)	40 (34–45)
≥ 70 years	57 (56–57)	24 (24–25)	63 (61–66)	22 (21–23)	67 (65–69)	34 (32–35)	67 (63–71)	22 (20–24)	70 (63–76)	19 (16–23)
12 months										
≤ 60 years	60 (58–61)	20 (20–21)	62 (59–65)	13 (12–15)	72 (69–76)	31 (28–34)	65 (58–71)	20 (17–23)	65 (56–72)	17 (13–22)
60–69 years	54 (52–55)	17 (17–18)	57 (54–59)	12 (11–13)	65 (62–68)	24 (22–26)	55 (49–60)	13 (11–15)	59 (50–66)	19 (15–24)
≥ 70 years	36 (35–36)	10 (10–11)	40 (38–42)	7 (7–8)	43 (40–45)	14 (13–15)	42 (37–46)	7 (6–9)	49 (42–56)	7 (5–10)
24 months										
≤ 60 years	36 (34–37)	7 (6–7)	31 (28–34)	4 (4–5)	47 (43–51)	10 (9–12)	39 (32–46)	7 (5–9)	35 (27–43)	6 (3–9)
60–69 years	30 (29–31)	5 (5–6)	30 (27–33)	3 (2–3)	38 (35–41)	6 (4–7)	30 (25–35)	3 (2–4)	24 (18–31)	4 (2–6)
≥ 70 years	18 (17–19)	3 (3–3)	20 (18–22)	2 (1–2)	21 (19–22)	3 (3–4)	19 (16–23)	2 (1–3)	25 (19–31)	2 (1–4)
36 months										
≤ 60 years	26 (24–27)	3 (3–4)	21 (18–24)	2 (2–3)	34 (30–37)	5 (4–7)	25 (19–32)	4 (2–6)	23 (16–30)	2 (1–4)
60–69 years	20 (19–21)	2 (2–3)	19 (17–22)	1 (1–2)	25 (22–28)	2 (1–3)	19 (14–24)	1 (< 1–2)	14 (9–20)	2 (1–3)
≥ 70 years	12 (11–12)	1 (1–2)	14 (12–16)	1 (< 1–1)	13 (11–14)	1 (1–2)	13 (10–17)	1 (< 1–1)	14 (9–19)	1 (< 1–2)
60 months										
≤ 60 years	19 (18–20)	2 (1–2)	14 (12–17)	1 (1–1)	23 (19–26)	2 (1–3)	16 (11–22)	1 (< 1–2)	15 (9–23)	2 (1–4)
60–69 years	13 (12–14)	1 (1–1)	12 (10–14)	< 1 (< 1–1)	16 (14–19)	1 (< 1–1)	13 (9–18)	< 1 (< 1–1)	8 (4–14)	1 (< 1–3)
≥ 70 years	7 (7–8)	1 (< 1–1)	7 (6–9)	< 1 (< 1–1)	7 (6–8)	1 (< 1–1)	8 (5–11)	< 1 (< 1–1)	8 (5–13)	< 1 (< 1–1)

*OS* overall survival, *CI* confidence interval

^a^Data are shown as survival proportion (95% confidence interval) [%]

### Temporal survival trends in overall and resected PaCs by stage

Trends in 1-month to 5-year survival of patients diagnosed in 2003–2005, 2006–2008, and 2009–2011 are shown in Table 4 and Additional file 1: Figures S3–S7. Significant survival changes between 2003 and 2005 and 2009–2011 are detailed below.

**Table 4:** Unadjusted survival rates in overall and resected pancreatic cancer patients diagnosed in 2003–2005, 2006–2008, and 2009–2011

Survival	Period	Stage	USA		The Netherlands		Belgium		Norway		Slovenia	
			Overall	Resected	Overall	Resected	Overall	Resected	Overall	Resected	Overall	Resected
			OS (95% CI)^a^	OS (95% CI)	OS (95% CI)	OS (95% CI)	OS (95% CI)	OS (95% CI)	OS (95% CI)	OS (95% CI)	OS (95% CI)	OS (95% CI)
1 month	2003–2005	I–II	83 (82–84)	95 (94–96)	89 (87–91)	96 (93–97)	94 (92–96)	97 (94–98)	91 (88–94)	99 (94–100)	89 (84–93)	96 (90–98)
		III–IV	61 (60–62)	86 (82–89)	73 (71–75)	88 (74–95)	88 (85–90)	96 (86–99)	78 (75–80)	100 (100–100)	69 (65–74)	92 (71–98)
	2006–2008	I–II	84 (84–85)	96 (95–96)	92 (91–94)	97 (95–98)	95 (94–97)	97 (95–98)	90 (83–96)	99 (93–100)	93 (88–96)	99 (93–100)
		III–IV	62 (61–63)	89 (86–91)	77 (75–78)	100 (100–100)	86 (85–88)	98 (91–99)	79 (76–81)	96 (75–99)	75 (71–78)	97 (80–100)
	2009–2011	I–II	86 (85–87)	96 (96–97)	92 (90–93)	97 (96–98)	96 (95–97)	98 (97–99)	94 (91–96)	98 (95–99)	90 (85–94)	99 (94–100)
		III–IV	63 (62–64)	90 (88–92)	76 (75–78)	91 (81–96)	85 (84–87)	100 (100–100)	78 (75–80)	96 (77–100)	75 (72–79)	98 (86–100)
	Percent unit change,^b^ P	I–II	**+ 3, 0.006**	**+ 2, 0.004**	**+ 3, 0.021**	+ 1, 0.257	+ 2, 0.116	+ 1, 0.577	+ 3, 0.175	–1, 0.473	+ 1, 0.851	+ 4, 0.083
		III–IV	**+ 2, 0.002**	+ 4, 0.989	**+ 3, 0.001**	+ 3, 0.635	–2, 0.086	+4, 0.189	+ < 1, 0.972	−4, 0.301	**+ 6, 0.024**	+ 6, 0.229
3 months	2003–2005	I–II	72 (71–73)	91 (90–92)	74 (71–76)	92 (89–94)	86 (83–89)	93 (90–96)	76 (71–80)	98 (93–99)	76 (69–82)	87 (79–92)
		III–IV	42 (41–43)	74 (69–78)	42 (40–43)	84 (69–92)	66 (62–69)	93 (82–97)	44 (41–47)	87 (68–95)	36 (31–40)	58 (36–75)
	2006–2008	I–II	73 (72–74)	92 (91–92)	78 (76–81)	93 (91–95)	87 (85–89)	92 (90–94)	72 (67–76)	94 (86–97)	80 (73–86)	92 (85–96)
		III–IV	44 (43–44)	79 (76–82)	46 (44–47)	98 (88–100)	63 (61–65)	85 (95–91)	48 (46–51)	84 (63–94)	43 (39–47)	85 (67–93)
	2009–2011	I–II	76 (75–77)	93 (92–94)	80 (78–82)	93 (91–95)	89 (87–91)	94 (92–96)	82 (78–85)	96 (92–98)	76 (70–82)	97 (91–99)
		III–IV	45 (44–46)	81 (78–84)	45 (43–46)	85 (74–92)	63 (60–65)	93 (85–96)	47 (44–49)	86 (66–94)	44 (40–48)	76 (61–86)
	Percent unit change,^b^ P	I–-II	**+ 4, < 0.001**	**+ 2, < 0.001**	**+ 6, 0.001**	+ 1, 0.546	+ 3, 0.118	+ 1, 0.508	**+ 6, 0.035**	− 1, 0.562	+ < 1, 0.909	**+ 10, 0.005**
		III–IV	**+ 3, < 0.001**	**+ 7, 0.017**	**+ 3, 0.001**	+ 1, 0.831	−3, 0.087	– < 1, 0.972	+ 3, 0.225	−1, 0.890	**+ 8, 0.007**	+ 18, 0.085
12 months	2003–2005	I–II	38 (36–39)	64 (62–66)	33 (30–36)	64 (59–68)	52 (48–57)	62 (56–67)	34 (29–39)	66 (57–74)	38 (30–45)	51 (41–60)
		III–IV	11 (11–12)	34 (29–39)	8 (7–9)	49 (33–63)	19 (17–22)	45 (31–57)	8 (6–10)	37 (20–53)	5 (3–8)	13 (3–29)
	2006–2008	I–II	40 (39–41)	65 (64–67)	36 (33–38)	64 (60–68)	56 (53–60)	69 (65–73)	33 (29–38)	71 (60–79)	51 (43–59)	67 (57–76)
		III–IV	12 (12–13)	35 (31–39)	8 (7–9)	53 (38–64)	19 (17–21)	47 (36–57)	11 (10–13)	56 (35–73)	12 (10–15)	39 (23–55)
	2009–2011	I–II	44 (43–45)	70 (68–71)	45 (43–48)	69 (65–72)	55 (52–57)	68 (65–71)	44 (39–49)	77 (70–83)	42 (35–48)	60 (50–68)
		III–IV	14 (14–15)	47 (42–51)	9 (8–10)	41 (29–52)	20 (18–22)	53 (42–62)	10 (8–11)	46 (28–63)	11 (9–14)	17 (8–30)
	Percent unit change,^b^ P	I–II	**+ 6, < 0.001**	**+ 5, < 0.001**	**+ 12, < 0.001**	+ 5, 0.066	+ 3, 0.297	+ 6, 0.057	**+ 10, 0.003**	**+ 11, 0.025**	+ 4, 0.577	+ 9, 0.101
		III–IV	**+ 3, < 0.001**	**+ 13, < 0.001**	**+ 1, 0.001**	−8, 0.496	+ 1, 0.476	+ 8, 0.548	**+ 2, 0.027**	+ 10, 0.689	**+ 6, < 0.001**	+ 5, 0.238
Survival	Period	Stage	USA		The Netherlands		Belgium		Norway		Slovenia	
			Overall	Resected	Overall	Resected	Overall	Resected	Overall	Resected	Overall	Resected
			OS (95% CI)^1^	OS (95% CI)	OS (95% CI)	OS (95% CI)	OS (95% CI)	OS (95% CI)	OS (95% CI)	OS (95% CI)	OS (95% CI)	OS (95% CI)
36 months	2003–2005	I–II	13 (13–14)	27 (25–29)	8 (7–10)	18 (14–22)	17 (14–21)	24 (19–29)	12 (9–16)	28 (20–36)	9 (6–14)	11 (6–17)
		III–IV	2 (2–2)	7 (5–10)	1 (1–2)	14 (6–26)	2 (2–4)	8 (2–16)	1 (1–2)	10 (3–24)	1 (< 1–2)	NA
	2006–2008	I–II	14 (14–15)	29 (27–30)	12 (10–13)	24 (20–28)	21 (18–23)	28 (25–32)	10 (7–14)	32 (22–41)	19 (14–26)	27 (19–37)
		III–IV	2 (2–2)	9 (7–11)	1 (1–1)	11 (5–21)	2 (2–3)	7 (3–14)	2 (1–2)	24 (10–42)	2 (1–3)	12 (4–26)
	2009–2011	I–II	17 (16–18)	33 (31–34)	16 (15–18)	29 (26–32)	20 (18–22)	30 (26–33)	15 (12–18)	30 (23–37)	12 (8–16)	17 (11–24)
		III–IV	2 (2–2)	11 (9–14)	1 (1–1)	12 (6–21)	2 (1–3)	10 (5–16)	1 (1–2)	14 (5–30)	1 (< 1–1)	NA
	Percent unit change,^b^ P	I–II	**+4, < 0.001**	**+ 5, < 0.001**	**+ 8, < 0.001**	**+ 11, < 0.001**	+ 3, 0.076	**+ 5, 0.035**	**+ 2, 0.025**	+ 2, 0.205	+ 2, 0.468	+ 6, 0.069
		III–IV	**+ < 1, < 0.001**	**+ 5, < 0.001**	**+ < 1, 0.005**	−2, 0.886	−1, 0.285	+ 2, 0.317	– < 1, 0.062	+4, 0.785	**+ < 1, 0.002**	NA, NA
60 months	2003–2005	I–II	9 (8–10)	18 (17–20)	4 (3–5)	10 (7–13)	11 (8–14)	16 (12–21)	9 (6–12)	18 (12–25)	7 (3–11)	9 (5–15)
		III–IV	1 (1–1)	4 (2–6)	1 (< 1–1)	9 (3–20)	1 (< 1–2)	4 (1–11)	1 (< 1–1)	3 (< 1–15)	< 1 (< 1–1)	NA
	2006–2008	I–II	10 (9–11)	20 (19–21)	7 (6–9)	15 (12–19)	13 (11–15)	18 (15–21)	7 (4–9)	20 (12–28)	9 (5–14)	13 (7–21)
		III–IV	1 (1–1)	6 (5–9)	< 1 (< 1–1)	6 (2–14)	1 (1–2)	5 (2–11)	1 (< 1–1)	4 (< 1–17)	2 (1–3)	12 (4–26)
	2009–2011	I–II	NA	NA	10 (8–12)	18 (15–21)	12 (10–14)	18 (15–21)	10 (7–13)	20 (14–27)	8 (5–12)	11 (6–17)
		III–IV	NA	NA	1 (< 1–1)	12 (6–21)	1 (< 1–1)	2 (< 1–7)	< 1 (< 1–1)	NA	< 1 (< 1–1)	NA
	Percent unit change,^b^ P	I–II	NA, NA	NA, NA	**+ 6, < 0.001**	**+ 8, < 0.001**	+ 1, 0.109	+ 2, 0.090	**+ 1, 0.043**	+ 2, 0.262	+ 2, 0.469	+ 2, 0.113
		III–IV	NA, NA	NA, NA	**+ < 1, 0.004**	+ 3, 0.935	–< 1, 0.290	−2, 0.409	- < 1, 0.069	NA, NA	**+ < 1, 0.002**	NA, NA

*OS* overall survival, *NA* not available as follow-up was not long enough

^a^Data are shown as survival rate (95% confidence interval) [%]

^b^Percent changes are shown by comparing average survival of patients diagnosed in 2009–2011 to those in 2003–2005. Significant changes according to *P* values calculated using the log-rank test are highlighted in bold

#### Short-term survival

Significant increases in 1-month survival for overall PaC patients were observed in the USA and the Netherlands, with 3 and 3% units increase (UI) for stage I–II and 2 and 3 UI for stages III–IV tumors. In Slovenia, an increment by 6 UI in 1-month survival was observed among total stages III–IV cancer patients. For the subgroup of resected patients, a significant survival increase was only observed for stages I–II cancer patients in the USA (2 UI). Improvements in 3-month survival were generally larger and also significant among total patients in the USA and the Netherlands, with 4 and 6 UI in stages I–II and 3 and 3 UI in stages III–IV cancers, respectively. In Norway, an increment by 6 UI was observed in patients with stages I–II cancer. In Slovenia, a significant increasing trend by 8 UI persisted in stages III–IV cancers. Within the resected subgroup, significant increasing trends were observed in both stages I–II (2 UI) and III–IV cancer patients (7 UI) in the USA, and in patients with stages III–IV cancer (10 UI) in Slovenia.

#### Longer-term survival

While in all countries 1-year survival increased for patients with stages I–II PaC, increases were only significant in the USA (6 UI), the Netherlands (12 UI), and Norway (10 UI). For the subgroup of resected patients, again 1-year survival increased in all countries, but changes were only significant in the USA (5 UI) and Norway (11 UI). For overall stages III–IV patients, 1-year survival increased significantly in the USA (3 UI), the Netherlands (1 UI), Norway (2 UI), and Slovenia (6 UI). For resected stages III–IV PaC patients, significant increases were only observed in the USA (13 UI). Improvements in 3-year survival for total stages I–II PaC patients were generally smaller and significant in the USA (4 UI), the Netherlands (8 UI), and Norway (2 UI). For the subgroup of resected stages I–II patients, significant increases were observed in the USA (5 UI), the Netherlands (11 UI), and Belgium (5 UI). Changes in 3-year survival for stages III–IV PaC were minor and significant only in the USA (< 1 UI) and the Netherlands (< 1 UI). Significant changes for the subgroup of resected stages III–IV PaC patients were observed only in the USA (5 UI). Regarding 5-year survival, significant increases were observed only in patients with stages I–II cancers. Survival rates increased by 6 and 1 UI in the Netherlands and Norway, respectively, for overall patients, and by 8 UI in the Netherlands for the resected patients.

## Discussion

This comprehensive, multinational, large-scale, population-based investigation provided overall survival estimates for overall patients and those with resected PaC by TNM stage and age. Furthermore, temporal trends for overall and resected cancer patients with clearly resectable (stages I–II) nd mostly unresectable (stages III–IV) PaCs in four European countries and the USA were shown respectively. In both stages I–II and III–IV tumors, survival rates decreased obviously with increasing age. Limited but encouraging progress in survival over time was detected.

According to EUROCARE-5 [4, 14], overall, the 1-, 3-, and 5-year survival rates of patients with PaC diagnosed during 1999–2007 in Europe were only 26%, 9%, and 7%, respectively. For the European countries investigated in this study, the 1-year survival was 19–34% and 5-year survival was 4–11%. In the USA, 5-year survival was 7–10% [16, 17]. Stage- and treatment-specific survival was not provided by the previous reports [4, 14]. Our study provided more up-to-date estimates by including patients diagnosed during 2003–2014 and further showed survival by TNM stage and age. Survival decreased with advancing stage and age. It is important to provide stratified survival for clinical counseling.

Guidelines [7–11] state that localized PaCs (stages I–II) are mostly resectable, while T4/stage III and M1/stage IV diseases are largely unresectable. Our results showed that resected patients with stages I–II PaCs had higher survival estimates in all age groups compared to the commonly reported and widely available overall ones. For example, resected patients < 60 years had 3–19%, 1–13%, and 1–9% units higher 1-, 3-, and 5-year survival than overall across countries, respectively. These differences may reflect effects of both resection and selection of fitter patients for resection. Given that most patients would perceive a general PaC prognosis as dismal and thus feel extremely distressed, also generating great burdens to their caregivers, it would be important to show them the objective survival data, especially for the resected patients, potentially rebuilding hope of life.

Survival in patients with stages III–IV PaCs, the majority of the diagnosed cases, was much poorer than that in those with stages I–II tumors, especially in the long term. In locally advanced PaC, the average overall survival remains < 1 year [18], and in metastatic tumors, the median survival is < 6 months [19], with 5-year survival of only ~ 2% [5]. We showed that even for those < 60 years, the overall 3- and 5-year survival was as low as 2–5% and 1–4%, respectively. Most patients with stages III–IV PaCs are deemed unresectable [9, 11]. This may, however, improve in the years to come with the increasing use of FOLFIRINOX [20]. In many of the cases where patients with metastatic PaC underwent resection, the metastasis was unexpectedly detected only during surgery [21]. Although resection rates in advanced tumors were low, notably, in stages III–IV PaCs substantially higher survival was observed for resected compared to total patients in all age groups, and resected patients < 70 years could have a 3-year survival of 5–34%. Even in those ≥ 70 years old, higher survival estimates for the resected subgroup were observed (1-year, 16–42% vs. 5–14%; 3-year, 2–14% vs. 1%–1%). While this difference might again at least partly reflect patient selection, i.e., inclusion of healthier patients or those with more favorable tumor characteristics for resection, our results indicated that not all patients with stages III–IV PaC had such a dismal prognosis as suggested by the overall survival estimates. These strong differences again underline the importance of reporting respective outcomes for stratified resected patients for enhanced counseling of these patients.

The perioperative survival is noteworthy, especially for elderly patients. It is volume-dependent, and is mainly influenced by failure to rescue and surgical expertise [22]. While resection could be safely performed for some proportion of the typically more vulnerable elderly patients [23, 24], at the population level, we found that in stages III–IV PaCs, which are associated with poorer general status, the 1-month survival dropped from 94–99% for patients < 70 years to 81–96% for those ≥ 70 years, which was more dramatic compared to results for the stages I–II disease. Age was negatively associated with survival, necessitating it as a stratification factor when providing survival information. Increasing ages are associated with more frequent comorbidities and complications, decreasing the potential survival benefits of resection. However, some studies suggested that, compared to younger individuals, fit elderly patients might obtain similar survival benefits from resection [23, 24]. The better survival observed for the younger patients might be partly explained by the more aggressive therapeutic strategies applied, which might contribute to survival improvements in the fit elderly as well [4]. These possibilities highlight the importance of geriatric assessment before treatment.

No substantial survival changes (5-year, 5–6%) were reported in PaC in the EUROCARE-5 study [4] over the period 1999–2007. In the USA, 5-year survival improved from 6 to 8% between 1992 and 1996 and 2002–2006 [25] and from 8 to 12% between 2002 and 2004 and 2008–2010 [16]; especially for localized tumors, strong improvement in 5-year survival by 7% units from 1998 to 2003 was observed [17]. We observed modest but nevertheless encouraging improvements in survival in patients both with stages I–II and III–IV tumors from 2003 to 2005 to 2009–2011, which potentially reflects the advancement in surgical skill, technique, and perioperative care. In the USA, 3-year survival increased by 4% units in stages I–II PaCs overall, but only by < 1% units in stages III–IV cancers. For resected cancers, survival increased by 5% units among patients with stages I–II cancer. In Europe, 3-year survival for both overall and resected patients with stages I–II PaC increased in all investigated countries, and a large increment was observed in the Netherlands (overall, 8% units; resected, 11% units), where postoperative mortality is decreasing [26]. Notably, centralization agreements were implemented in the Netherlands since 2005, and promoted more resections [27], which might be associated with the continuous improvement in survival [28]. While further major survival advancement in resected patients could be limited even with surgical technique modification, improved outcomes are likely to come from more effective systemic treatments (e.g., FOLFIRINOX) combined with surgery. The different trends between overall and resected patients further highlight the need to offer survival data in specific subgroups.

This study covered the periods when the sixth and seventh TNM staging systems were in effect, and both are compatible/identical with each other [8]. While potentially improved imaging might result in a shift in stage classification, the proportions of each stage remained relatively stable in investigated countries (data not shown). In the era of the eighth TNM staging, where the definitions of the T4 and M1 categories indicating mostly unresectable cancers remain unchanged [29], our results would still be usable for prognosis counseling.

This study was limited by the small case numbers in some subgroups. Further potentially prognostically important factors (e.g., comorbidity) were not considered because they were unknown/unavailable in the national registries of most countries. Although older ages and more advanced tumor stages herein investigated were the most prominent negative prognostic factors and might contraindicate resection, precise and personalized factors should be considered for evaluation of an individual patient’s prognosis. Some prognostic tools (e.g., the nomogram) might offer more precise prognostic information for a specific patient. Non-surgical treatment was not incorporated considering the low sensitivity in recording in some registries and the varying regimens used. Information from more countries would increase the comprehensiveness of the report. However, treatment or TNM staging data were mostly not readily available in the other national population-based registries. Our study was based on complete-case analysis. Differences in data recording, especially of TNM stage, should be noted, and the stages I–II cancer proportion varied from 25% in Norway and Slovenia to 38% in Belgium. There could be underreporting, especially of advanced-stage disease, with various extents, besides the potential impact of missing staging information. These differences highlight the need for standardization in the registration practice. Potential variation in registration practice, especially for stage, might affect outcomes, and inter-country comparisons were not made considering the possible heterogeneity. Results were only analyzed and interpreted independently in the respective country without pooling or comparison with other countries. Results from a specific national registry might not be generalizable to another country. For counseling of patients not in the studied countries, other aspects including treatment profiles and health care systems should be considered.

In the main analyses, we included PaC cases irrespective of microscopic confirmation, which is in accordance with the real-world situation [15] and which is also consistent with the approach in the EUROCARE studies [4, 14]. While resected cases were mostly microscopically confirmed, the confirmation rates for overall cases varied. The microscopic confirmation rates for PaC have been relatively low [4], and PaC has always been difficult to verify microscopically, especially unresectable PaC. In our complete-case study inclusion of patients with known stage might have an impact on the observed confirmation rates. After limiting the overall cases to the microscopically confirmed ones in sensitivity analyses, the survival estimates mostly became higher in all participating countries except in Belgium, where microscopic confirmation rates were high. Furthermore, the survival increase was the most prominent in patients ≥ 70 years old, who are generally more frail and for whom the selection of treatment is usually more cautious. While including microscopically confirmed cases only could help to further increase the chance of selecting real PaC patients, those not receiving any treatment and usually having poorer patient and/or tumor characteristics might be more likely excluded, potentially partly explaining the higher observed survival estimates in the sensitivity analyses.

We showed that it is important to provide survival estimates separately to resected patients for counseling, as the resected subgroup has substantially higher survival than the overall estimation. We did not show the results for unresected PaC patients and avoided direct comparisons between the resected and the unresected, as they may to a large extent reflect selection effects related to factors including patients’ health status and hospital characteristics. In the resected subgroup, curative and palliative resections were not differentiated from each other, considering the greatly geographically and temporally varying standards for defining clear resection margins in PaC surgery.

Nevertheless, the multinational, population-based, large-scale character of this study with the country-specific respective analysis adds important novel survival data to the literature. In particular, results stratified by TNM stage and age for resected and overall patients will further aid patient counseling in clinical practice, providing more specific survival information for specific patient populations.

## Conclusions

Our international population-based study provides comprehensive data on survival expectations of resected PaC patients which are substantially higher than the widely available and known dismal survival prognosis for total patients. The benefits of resection cannot be concluded from this observational study. However, the TNM stage- and age-stratified survival results might be helpful for clinical counseling. Estimated survival for advanced-stage disease should be interpreted with caution due to potential underreporting. Patients with advanced stage and/or old age should undergo careful assessment. Limited but encouraging survival improvement is observed.

## Additional file

Additional file 1: **Table S1.** Selection of contacted national population-based cancer registries in Europe. **Table S2.** General information on participating population-based registries. **Table S3.** Inclusion codes according to International Classification of Diseases for Oncology, Third Edition. **Table S4.** Unadjusted survival proportions in patients with overall and resected stages III–IV PaC. **Figure S1.** Kaplan-Meier curves (*solid lines*) of age group-specific survival in overall (*upper panel*) and resected patients (*lower panel*) with TNM stages III–IV pancreatic cancers. The *dashed lines* indicate the 95% confidence limits, and the *shadows* represent the Hall-Wellner confidence bands. Numbers of patients at risk are also reported. Median survival is in months. *IQR* interquartile range. **Figure S2.** Kaplan-Meier curves (*solid lines*) of age group-specific survival in microscopically confirmed overall TNM stages I–II (*upper panel*) and stages III–IV pancreatic cancer patients (*lower panel*). The *dashed lines* indicate the 95% confidence limits, and the *shadows* represent the Hall-Wellner confidence bands. Numbers of patients at risk are also reported. Median survival is in months. *IQR* interquartile range. **Figure S3.** Changes in 1-month survival over calendar periods among overall and resected patients with stages I–II and III–IV pancreatic cancers. **Figure S4.** Changes in 3-month survival over calendar periods among overall and resected patients with stages I–II and III–IV pancreatic cancers. **Figure S5.** Changes in 12-month survival over calendar periods among overall and resected patients with stages I–II and III–IV pancreatic cancers. **Figure S6.** Changes in 36-month survival over calendar periods among overall and resected patients with stages I–II and III–IV pancreatic cancers. **Figure S7.** Changes in 60-month survival over calendar periods among overall and resected patients with stages I–II and III–IV pancreatic cancers. **Supplementary Results.** Patient characteristics. (DOCX 3259 kb)

## Abbreviations

AJCC: American Joint Committee on Cancer
NCCN: National Comprehensive Cancer Network
PaC: Pancreatic cancer
SEER: Surveillance, Epidemiology, and End Results
UICC: Union for International Cancer Control

## References

[1] Torre LA, Bray F, Siegel RL, Ferlay J, Lortet-Tieulent J, Jemal A (2015). Global cancer statistics, 2012. CA Cancer J Clin.

[2] Malvezzi M, Carioli G, Bertuccio P, Rosso T, Boffetta P, Levi F, La Vecchia C, Negri E (2016). European cancer mortality predictions for the year 2016 with focus on leukaemias. Ann Oncol.

[3] Ferlay J, Partensky C, Bray F (2016). More deaths from pancreatic cancer than breast cancer in the EU by 2017. Acta Oncol.

[4] Lepage C, Capocaccia R, Hackl M, Lemmens V, Molina E, Pierannunzio D, Sant M, Trama A, Faivre J, Grouph E-W (2015). Survival in patients with primary liver cancer, gallbladder and extrahepatic biliary tract cancer and pancreatic cancer in Europe 1999−2007: results of EUROCARE-5. Eur J Cancer.

[5] Wolfgang CL, Herman JM, Laheru DA, Klein AP, Erdek MA, Fishman EK, Hruban RH (2013). Recent progress in pancreatic cancer. CA Cancer J Clin.

[6] Ryan DP, Hong TS, Bardeesy N (2014). Pancreatic adenocarcinoma. N Engl J Med.

[7] Tempero MA, Malafa MP, Behrman SW, Benson AB, Casper ES, Chiorean EG, Chung V, Cohen SJ, Czito B, Engebretson A (2014). Pancreatic adenocarcinoma, version 2.2014: featured updates to the NCCN guidelines. J Natl Compr Cancer Netw.

[8] Ducreux M, Cuhna AS, Caramella C, Hollebecque A, Burtin P, Goere D, Seufferlein T, Haustermans K, Van Laethem JL, Conroy T (2015). Cancer of the pancreas: ESMO Clinical Practice Guidelines for diagnosis, treatment and follow-up. Ann Oncol.

[9] Sohal DP, Mangu PB, Khorana AA, Shah MA, Philip PA, O'Reilly EM, Uronis HE, Ramanathan RK, Crane CH, Engebretson A (2016). Metastatic pancreatic cancer: American Society of Clinical Oncology Clinical Practice Guideline. J Clin Oncol.

[10] Khorana AA, Mangu PB, Berlin J, Engebretson A, Hong TS, Maitra A, Mohile SG, Mumber M, Schulick R, Shapiro M (2016). Potentially curable pancreatic Cancer: American Society of Clinical Oncology Clinical Practice Guideline. J Clin Oncol.

[11] Balaban EP, Mangu PB, Khorana AA, Shah MA, Mukherjee S, Crane CH, Javle MM, Eads JR, Allen P, Ko AH (2016). Locally advanced, unresectable pancreatic cancer: American Society of Clinical Oncology Clinical Practice Guideline. J Clin Oncol.

[12] Evans DB, George B, Tsai S (2015). Non-metastatic pancreatic cancer: resectable, borderline resectable, and locally advanced-definitions of increasing importance for the optimal delivery of multimodality therapy. Ann Surg Oncol.

[13] Huang L, Jansen L, Balavarca Y, Molina-Montes E, Babaei M, van der Geest L, Lemmens V, Van Eycken L, De Schutter H, Johannesen TB et al.: Resection of pancreatic cancer in Europe and USA: an international large-scale study highlighting large variations. Gut 2017. 10.1136/gutjnl-2017-314828. https://www.ncbi.nlm.nih.gov/pubmed/29158237. Accessed 10 Jan 2018. 10.1136/gutjnl-2017-314828 29158237

[14] De Angelis R, Sant M, Coleman MP, Francisci S, Baili P, Pierannunzio D, Trama A, Visser O, Brenner H, Ardanaz E (2014). Cancer survival in Europe 1999-2007 by country and age: results of EUROCARE--5-a population-based study. Lancet Oncol.

[15] Asbun HJ, Conlon K, Fernandez-Cruz L, Friess H, Shrikhande SV, Adham M, Bassi C, Bockhorn M, Buchler M, Charnley RM (2014). When to perform a pancreatoduodenectomy in the absence of positive histology? A consensus statement by the International Study Group of Pancreatic Surgery. Surgery.

[16] Sirri E, Castro FA, Kieschke J, Jansen L, Emrich K, Gondos A, Holleczek B, Katalinic A, Urbschat I, Vohmann C (2016). Recent trends in survival of patients with pancreatic Cancer in Germany and the United States. Pancreas.

[17] Brenner H, Gondos A, Arndt V (2007). Recent major progress in long-term cancer patient survival disclosed by modeled period analysis. J Clin Oncol.

[18] Loehrer PJ, Feng Y, Cardenes H, Wagner L, Brell JM, Cella D, Flynn P, Ramanathan RK, Crane CH, Alberts SR (2011). Gemcitabine alone versus gemcitabine plus radiotherapy in patients with locally advanced pancreatic cancer: an Eastern Cooperative Oncology Group trial. J Clin Oncol.

[19] Hammel P, Huguet F, van Laethem JL, Goldstein D, Glimelius B, Artru P, Borbath I, Bouche O, Shannon J, Andre T (2016). Effect of chemoradiotherapy vs chemotherapy on survival in patients with locally advanced pancreatic cancer controlled after 4 months of gemcitabine with or without erlotinib: the LAP07 randomized clinical trial. JAMA.

[20] Suker M, Beumer BR, Sadot E, Marthey L, Faris JE, Mellon EA, El-Rayes BF, Wang-Gillam A, Lacy J, Hosein PJ (2016). FOLFIRINOX for locally advanced pancreatic cancer: a systematic review and patient-level meta-analysis. Lancet Oncol.

[21] Kim Y, Kim SC, Song KB, Kim J, Kang DR, Lee JH, Park KM, Lee YJ (2016). Improved survival after palliative resection of unsuspected stage IV pancreatic ductal adenocarcinoma. HPB (Oxford).

[22] Krautz C, Nimptsch U, Weber GF, Mansky T, Grutzmann R (2018). Effect of hospital volume on in-hospital morbidity and mortality following pancreatic surgery in Germany. Ann Surg.

[23] van der Geest LG, Besselink MG, van Gestel YR, Busch OR, de Hingh IH, de Jong KP, Molenaar IQ, Lemmens VE (2016). Pancreatic cancer surgery in elderly patients: balancing between short-term harm and long-term benefit. A population-based study in the Netherlands. Acta Oncol.

[24] Barbas AS, Turley RS, Ceppa EP, Reddy SK, Blazer DG, Clary BM, Pappas TN, Tyler DS, White RR, Lagoo SA (2012). Comparison of outcomes and the use of multimodality therapy in young and elderly people undergoing surgical resection of pancreatic cancer. J Am Geriatr Soc.

[25] Pulte D, Redaniel MT, Brenner H, Jeffreys M (2012). Changes in survival by ethnicity of patients with cancer between 1992-1996 and 2002-2006: is the discrepancy decreasing?. Ann Oncol.

[26] de Wilde RF, Besselink MG, van der Tweel I, de Hingh IH, van Eijck CH, Dejong CH, Porte RJ, Gouma DJ, Busch OR, Molenaar IQ (2012). Impact of nationwide centralization of pancreaticoduodenectomy on hospital mortality. Br J Surg.

[27] Lemmens VE, Bosscha K, van der Schelling G, Brenninkmeijer S, Coebergh JW, de Hingh IH (2011). Improving outcome for patients with pancreatic cancer through centralization. Br J Surg.

[28] van der Geest LG, van Rijssen LB, Molenaar IQ, de Hingh IH, Groot Koerkamp B, Busch OR, Lemmens VE, Besselink MG (2016). Volume-outcome relationships in pancreatoduodenectomy for cancer. HPB (Oxford).

[29] Shi S, Hua J, Liang C, Meng Q, Liang D, Xu J, Ni Q, Yu X. Proposed modification of the 8th edition of the AJCC staging system for pancreatic ductal adenocarcinoma. Ann Surg. 2018; 10.1097/SLA.000000000002668. 10.1097/SLA.0000000000002668 29334560

