# Supplementary material for: Stratified survival of resected and overall pancreatic cancer patients in Europe and the USA in the early twenty-first century: a large, international population-based study
Source: BMC Med. 2018 Aug 21;16:125. doi: 10.1186/s12916-018-1120-9 (PMC6102804; doi:10.1186/s12916-018-1120-9)
Supplement: Supplementary file 1 — Table S1. Selection of contacted national population-based cancer registries in Europe. Table S2. General information on participating population-based registries. Table S3. Inclusion codes according to International Classification of Diseases for Oncology, Third Edition. Table S4. Unadjusted survival proportions in patients with overall and resected stages III–IV PaC. Figure S1. Kaplan-Meier curves (solid lines) of age group-specific survival in overall (upper panel) and resected patients (lower panel) with TNM stages III–IV pancreatic cancers. The dashed lines indicate the 95% confidence limits, and the shadows represent the Hall-Wellner confidence bands. Numbers of patients at risk are also reported. Median survival is in months. IQR interquartile range. Figure S2. Kaplan-Meier curves (solid lines) of age group-specific survival in microscopically confirmed overall TNM stages I–II (upper panel) and stages III–IV pancreatic cancer patients (lower panel). The dashed lines indicate the 95% confidence limits, and the shadows represent the Hall-Wellner confidence bands. Numbers of patients at risk are also reported. Median survival is in months. IQR interquartile range. Figure S3. Changes in 1-month survival over calendar periods among overall and resected patients with stages I–II and III–IV pancreatic cancers. Figure S4. Changes in 3-month survival over calendar periods among overall and resected patients with stages I–II and III–IV pancreatic cancers. Figure S5. Changes in 12-month survival over calendar periods among overall and resected patients with stages I–II and III–IV pancreatic cancers. Figure S6. Changes in 36-month survival over calendar periods among overall and resected patients with stages I–II and III–IV pancreatic cancers. Figure S7. Changes in 60-month survival over calendar periods among overall and resected patients with stages I–II and III–IV pancreatic cancers. Supplementary Results. Patient characteristics. (DOCX 3259 kb) [file 12916_2018_1120_MOESM1_ESM.docx]

**Supplementary materials**

**Supplementary Results.** Patient characteristics

**Table S1.** Selection of contacted national population-based cancer registries in Europe

**Table S2.** General information on participating population-based registries

**Table S3.** Inclusion codes according to International Classification of Diseases for Oncology, Third Edition

**Table S4.** Unadjusted survival proportions in overall and resected stage III-IV pancreatic cancer patients

**Figure S1.** Kaplan-Meier curves (solid lines) of age group-specific survival in overall (upper panel) and resected patients (lower panel) with TNM stage III-IV pancreatic cancers. The dashed lines indicate the 95% confidence limits and the shadows represent the Hall-Wellner confidence bands. Numbers of patients at risk are also reported. Median survival is in months. IQR, interquartile range.

**Figure S2.** Kaplan-Meier curves (solid lines) of age group-specific survival in microscopically confirmed overall TNM stage I-II (upper panel) and stage III-IV pancreatic cancer patients (lower panel). The dashed lines indicate the 95% confidence limits and the shadows represent the Hall-Wellner confidence bands. Numbers of patients at risk are also reported. Median survival is in months. IQR, interquartile range.

**Figure S3.** Changes in 1-month survival over calendar periods among overall and resected patients with stages I-II and III-IV pancreatic cancers

**Figure S4.** Changes in 3-month survival over calendar periods among overall and resected patients with stages I-II and III-IV pancreatic cancers

**Figure S5.** Changes in 12-month survival over calendar periods among overall and resected patients with stages I-II and III-IV pancreatic cancers

**Figure S6.** Changes in 36-month survival over calendar periods among overall and resected patients with stages I-II and III-IV pancreatic cancers

**Figure S7.** Changes in 60-month survival over calendar periods among overall and resected patients with stages I-II and III-IV pancreatic cancers

**Supplementary Results**

***Patient characteristics***

In stage I-II PaCs, 49%-55% of the overall patients were female. The mean age was 69-72 years. Most tumors were located in pancreas head (81%-89%). Only 10%-18% of the PaCs were well-differentiated. Chemotherapy was administered for 17% (Norway) to 52% (Belgium) of the patients in Europe. Radiotherapy was more often applied in USA (14%) than in Europe (1% (Slovenia) to 10% (Belgium)). Resection rates were 34% (Norway) to 63% (Belgium). Compared to overall patients, operated patients were less often female and generally younger. Tumor location was comparable, but slightly fewer tumors were well-differentiated (6%-17%). Neoadjuvant chemotherapy (1%-3%) and radiotherapy (0%-4%) were rarely administered. Resected patients received more often adjuvant chemotherapy (24%-56%), but less frequently radiotherapy (1%-9%) compared to the whole group in Europe. In USA, resected patients received markedly more frequently radiotherapy (32%) compared to the overall US or the resected European patients.

Compared to those with stage I-II PaCs, overall patients with stage III-IV tumors were less often women (48%-50%), and were younger (mean age, 68-71 years). Pancreas head tumors comprised smaller proportions (56%-64%), and well-differentiated cancers were rarer (8%-17%). Chemotherapy was more frequently administered (18% (Slovenia) to 65% (Belgium)), while radiotherapy was less commonly applied (1% (USA) to 6% (Belgium)). Resection rates were 2% (the Netherlands) to 7% (Slovenia). The comparison patterns of resected versus overall patients with stage III-IV PaCs were similar to those with stage I-II cancers in sex, age, tumor differentiation, and chemotherapy application. However, the proportions of pancreas head tumors among resected patients were greater (66%-79%). Neoadjuvant chemotherapy (0%-8%) and radiotherapy rates (0%-7%) remained low. Radiotherapy was applied in 3%-19% of resected patients, and was again more often used in USA.

**Table S1.** Selection of contacted national population-based cancer registries in Europe^1^

| Country of contacted registry | Included | Comment if not included |
| --- | --- | --- |
| *Norther Europe* |  |  |
| Finland | No | Surgical treatment not validated |
| Sweden | No | National data not statistically validated |
| Norway | Yes |  |
| Iceland | No | No national population-based data on treatment |
| Denmark | No | Participation consent withdrawn due to legislation issues |
| *Eastern Europe* |  |  |
| Estonia | No | Small number of resected cases and short incidence periods recorded not allowing for robust survival analysis |
| Latvia | No | No national population-based data on treatment |
| Lithuania | No | No response |
| Ukraine | No | Insufficient resources for data collection |
| Slovakia | No | No response |
| *Central Europe* |  |  |
| Poland | No | No response |
| Czech Republic | No | No national population-based data on treatment |
| Austria | No | No national population-based data on treatment |
| *Western Europe* |  |  |
| The UK | No | No ready-to-use national population-based data on treatment or TNM stage |
| Ireland | No | No further response after initial contact |
| The Netherlands | Yes |  |
| Belgium | Yes |  |
| *Southern Europe* |  |  |
| Bulgaria | No | No national population-based data on treatment |
| Serbia | No | No response |
| Slovenia | Yes |  |
| Croatia | No | No national population-based data on surgical treatment |

^1^For the other countries and regions in Europe (*e.g.*, Italy, Germany, and France) not listed in this table, no corresponding national population-based registries were found by us through careful search.

**Table S2.** General information on participating population-based registries

| Source | Country | Diagnosis period | Censoring date | Registered malignant cases | Excluded cases^1^ | | |  | Analyzed cases | |
| --- | --- | --- | --- | --- | --- | --- | --- | --- | --- | --- |
|  |  |  |  |  | DCO/autopsy | Unknown stage/stage 0 | Unknown survival^2^ |  | Stage I-II | Stage III-IV |
| SEER18^3^ | USA | Jan. 2004-  Dec. 2013 | Dec. 31, 2013 | 99582 | 2972 (3) | 10144 (10) | 0 (0) |  | 31313 | 55153 |
| NCR | The Netherlands | Jan. 2003-  Dec. 2014 | Feb 1, 2015 | 22579 | 99 (<1) | 2796 (12) | 0 (0) |  | 5710 | 13974 |
| BCR | Belgium | Jan. 2004-  Dec. 2013 | Jul. 1, 2015 | 12146 | -^4^ | 3077 (25) | 0 (0) |  | 3437 | 5632 |
| CRN | Norway | Jan. 2003-  Dec. 2014 | Jun. 30, 2015 | 8024 | 333 (4) | 1511 (19) | 2 (<1) |  | 1545 | 4633 |
| CRS | Slovenia | Jan. 2003-  Dec. 2013 | May 25, 2016 | 3376 | 54 (2) | 658 (20) | 0 (0) |  | 667 | 1997 |

^1^Shown as n (percentage [%]).

^2^Unknown survival time and/or vital status.

^3^Data of the year 2003 was not analyzed, as the TNM stage (version 6/7) information was unavailable.

^4^Not routinely registered.

SEER, [Surveillance, Epidemiology, and End Results Program](http://seer.cancer.gov/); NCR, The Netherlands Cancer Registry; BCR, Belgian Cancer Registry; CRN, Cancer Registry of Norway; CRS, Cancer Registry of Slovenia; DCO, death certificate only.

**Table S3.** Inclusion codes according to International Classification of Diseases for Oncology, Third Edition

| **Category** | **Code** |
| --- | --- |
| **Topography** | C25.0, C25.1, C25.2, C25.3, C25.7, C25.8, C25.9 |
| **Morphology**^1^ | 8000-8009, 8010-8012 & 8014-8049, 8050-8089, 8140-8149, 8154, 8158, 8159, 8161, 8163-8169, 8171-8179, 8181-8239, 8244-8245, 8250-8311 & 8313-8389, 8440-8499, 8500-8549, 8550-8559, 8560-8579 |
| **Behavior** | 3 |

**Table S4.** Unadjusted survival proportions in overall and resected stage III-IV pancreatic cancer patients

| **Time** | **The US** | | **The Netherlands** | | **Belgium** | | **Norway** | | **Slovenia** | |
| --- | --- | --- | --- | --- | --- | --- | --- | --- | --- | --- |
|  | ***Overall*** | ***Resected***^2^ | ***Overall*** | ***Resected*** | ***Overall*** | ***Resected*** | ***Overall*** | ***Resected*** | ***Overall*** | ***Resected*** |
|  | OS (95% CI)^1^ | OS (95% CI) | OS (95% CI) | OS (95% CI) | OS (95% CI) | OS (95% CI) | OS (95% CI) | OS (95% CI) | OS (95% CI) | OS (95% CI) |
| *1 month* |  |  |  |  |  |  |  |  |  |  |
| < 60 years | 75 (74-76) | 94 (93-96) | 86 (85-87) | 94 (89-97) | 93 (92-95) | 99 (95-100) | 91 (89-93) | 97 (90-99) | 83 (79-86) | 99 (92-100) |
| 60-69 years | 69 (68-70) |  | 81 (80-82) |  | 89 (88-91) |  | 84 (82-86) |  | 79 (75-82) |  |
| ≥ 70 years | 53 (53-54) | 81 (78-84) | 69 (68-70) | 96 (88-99) | 81 (80-83) | 96 (90-99) | 73 (71-74) | 91 (74-97) | 67 (64-70) | 90 (78-96) |
| *3 months* |  |  |  |  |  |  |  |  |  |  |
| < 60 years | 58 (57-58) | 86 (84-88) | 59 (57-61) | 90 (84-93) | 78 (75-80) | 93 (89-96) | 65 (62-68) | 89 (80-95) | 55 (50-60) | 81 (71-88) |
| 60-69 years | 51 (50-52) |  | 52 (50-53) |  | 69 (67-71) |  | 57 (54-59) |  | 49 (44-53) |  |
| ≥ 70 years | 34 (34-35) | 68 (64-71) | 36 (35-37) | 89 (79-94) | 55 (53-56) | 85 (77-91) | 37 (35-39) | 76 (57-87) | 33 (30-35) | 68 (53-79) |
| *6 months* |  |  |  |  |  |  |  |  |  |  |
| < 60 years | 40 (39-41) | 71 (68-73) | 34 (32-36) | 76 (68-82) | 59 (56-62) | 85 (79-89) | 41 (38-45) | 72 (60-81) | 38 (33-42) | 68 (57-77) |
| 60-69 years | 34 (33-35) |  | 29 (28-31) |  | 50 (48-53) |  | 35 (32-38) |  | 32 (28-36) |  |
| ≥ 70 years | 21 (20-21) | 53 (49-56) | 18 (17-18) | 72 (60-82) | 34 (32-35) | 66 (56-74) | 18 (16-19) | 49 (31-64) | 14 (12-16) | 42 (28-55) |
| *12 months* |  |  |  |  |  |  |  |  |  |  |
| < 60 years | 20 (19-21) | 47 (44-50) | 13 (11-14) | 48 (40-56) | 31 (28-34) | 57 (50-64) | 19 (16-22) | 45 (33-56) | 14 (11-18) | 32 (22-42) |
| 60-69 years | 16 (16-17) |  | 11 (10-12) |  | 24 (22-26) |  | 13 (11-14) |  | 15 (12-18) |  |
| ≥ 70 years | 9 (9-9) | 32 (28-35) | 6 (5-7) | 42 (30-53) | 14 (13-15) | 36 (27-45) | 6 (5-7) | 39 (22-55) | 5 (4-7) | 16 (8-27) |
| *24 months* |  |  |  |  |  |  |  |  |  |  |
| < 60 years | 7 (6-7) | 21 (19-24) | 4 (3-5) | 21 (15-28) | 10 (9-12) | 23 (17-29) | 7 (5-9) | 25 (15-35) | 5 (3-7) | 11 (5-18) |
| 60-69 years | 5 (5-6) |  | 2 (2-3) |  | 6 (5-7) |  | 3 (2-4) |  | 3 (1-4) |  |
| ≥ 70 years | 3 (2-3) | 13 (11-16) | 1 (1-2) | 19 (10-30) | 4 (3-4) | 12 (6-19) | 2 (1-2) | 19 (7-34) | 1 (1-2) | 4 (1-12) |
| *36 months* |  |  |  |  |  |  |  |  |  |  |
| < 60 years | 3 (3-4) | 11 (9-13) | 2 (2-3) | 11 (7-17) | 5 (4-7) | 12 (8-17) | 4 (3-6) | 19 (11-29) | 2 (1-4) | 5 (2-11) |
| 60-69 years | 2 (2-2) |  | 1 (1-1) |  | 2 (1-3) |  | 1 (1-2) |  | 1 (<1-2) |  |
| ≥ 70 years | 1 (1-1) | 8 (5-10) | 1 (1-1) | 14 (7-24) | 1 (1-2) | 5 (2-11) | 1 (1-1) | 7 (1-21) | 1 (<1-1) | 2 (<1-9) |
| *60 months* |  |  |  |  |  |  |  |  |  |  |
| < 60 years | 2 (2-2) | 8 (6-10) | 1 (1-1) | 9 (5-14) | 2 (1-4) | 4 (2-8) | 1 (1-3) | 3 (1-10) | 1 (1-3) | 4 (1-9) |
| 60-69 years | 1 (1-1) |  | 1 (<1-1) |  | 1 (<1-1) |  | 1 (<1-1) |  | 1 (<1-2) |  |
| ≥ 70 years | 1 (1-1) | 5 (3-7) | <1 (<1-1) | 9 (3-19) | 1 (<1-1) | 4 (1-10) | <1 (<1-1) | NA | <1 (<1-1) | NA |

^1^Data are shown as survival proportion (95% confidence interval) [%].

^2^For the resected group in each center, the age groups ‘< 60 years’ and ‘60-69 years’ were combined to the group ‘< 70 years’ due to limited case number in either.

OS, overall survival; CI, confidence interval; NA, not available.


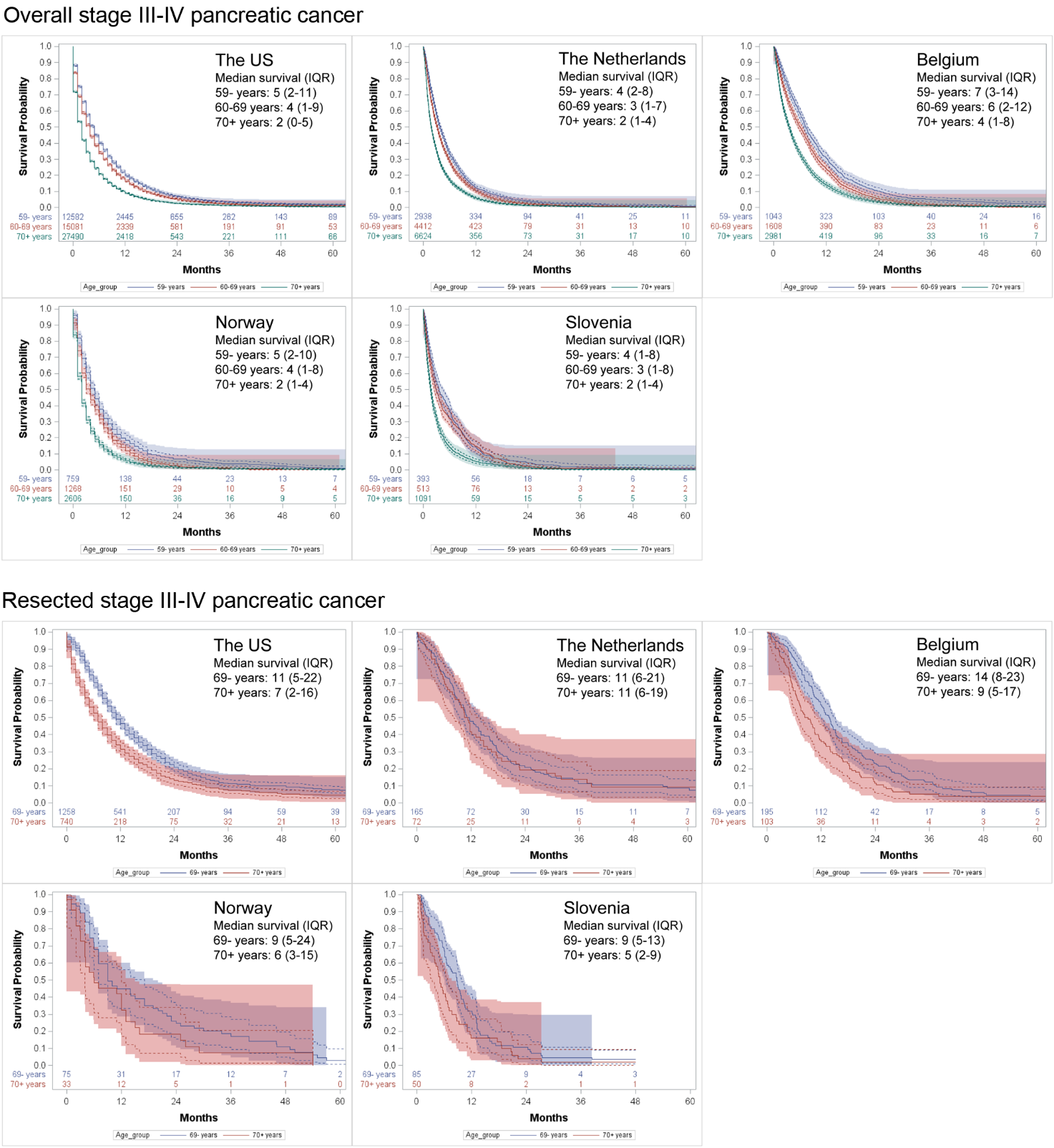


**Figure S1. Kaplan-Meier curves (solid lines) of age group-specific survival in overall (upper panel) and resected patients (lower panel) with TNM stage III-IV pancreatic cancers.** The dashed lines indicate the 95% confidence limits and the shadows represent the Hall-Wellner confidence bands. Numbers of patients at risk are also reported. Median survival is in months. IQR, interquartile range.

Overall stage I-II pancreatic cancer (microscopically confirmed)


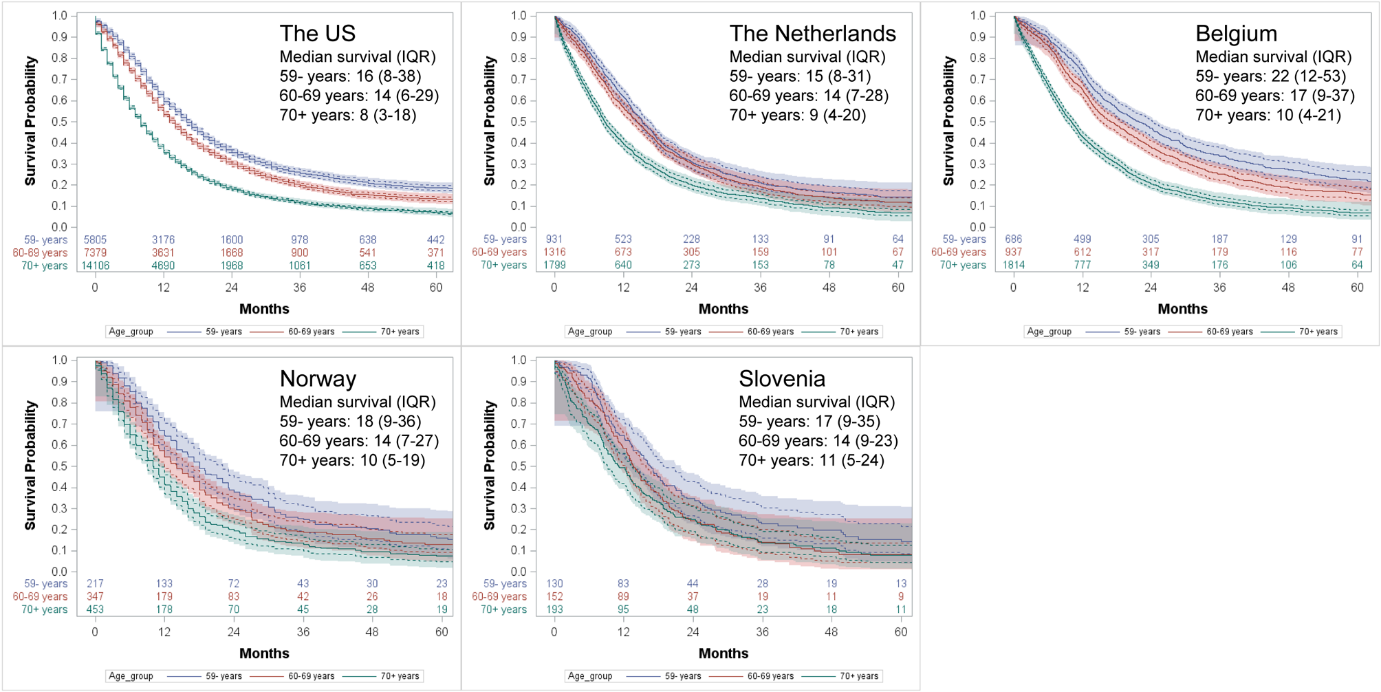


Overall stage III-IV pancreatic cancer (microscopically confirmed)


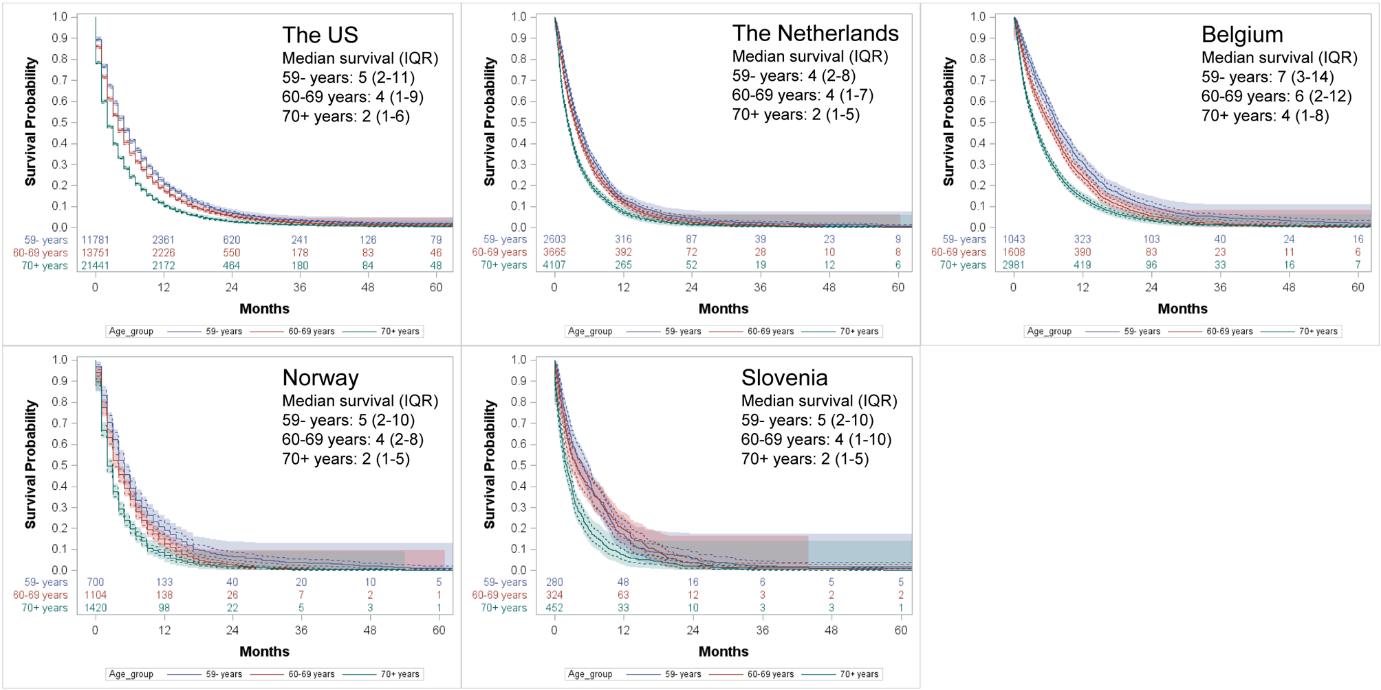


**Figure S2. Kaplan-Meier curves (solid lines) of age group-specific survival in microscopically confirmed overall TNM stage I-II (upper panel) and stage III-IV pancreatic cancer patients (lower panel).** The dashed lines indicate the 95% confidence limits and the shadows represent the Hall-Wellner confidence bands. Numbers of patients at risk are also reported. Median survival is in months. IQR, interquartile range.


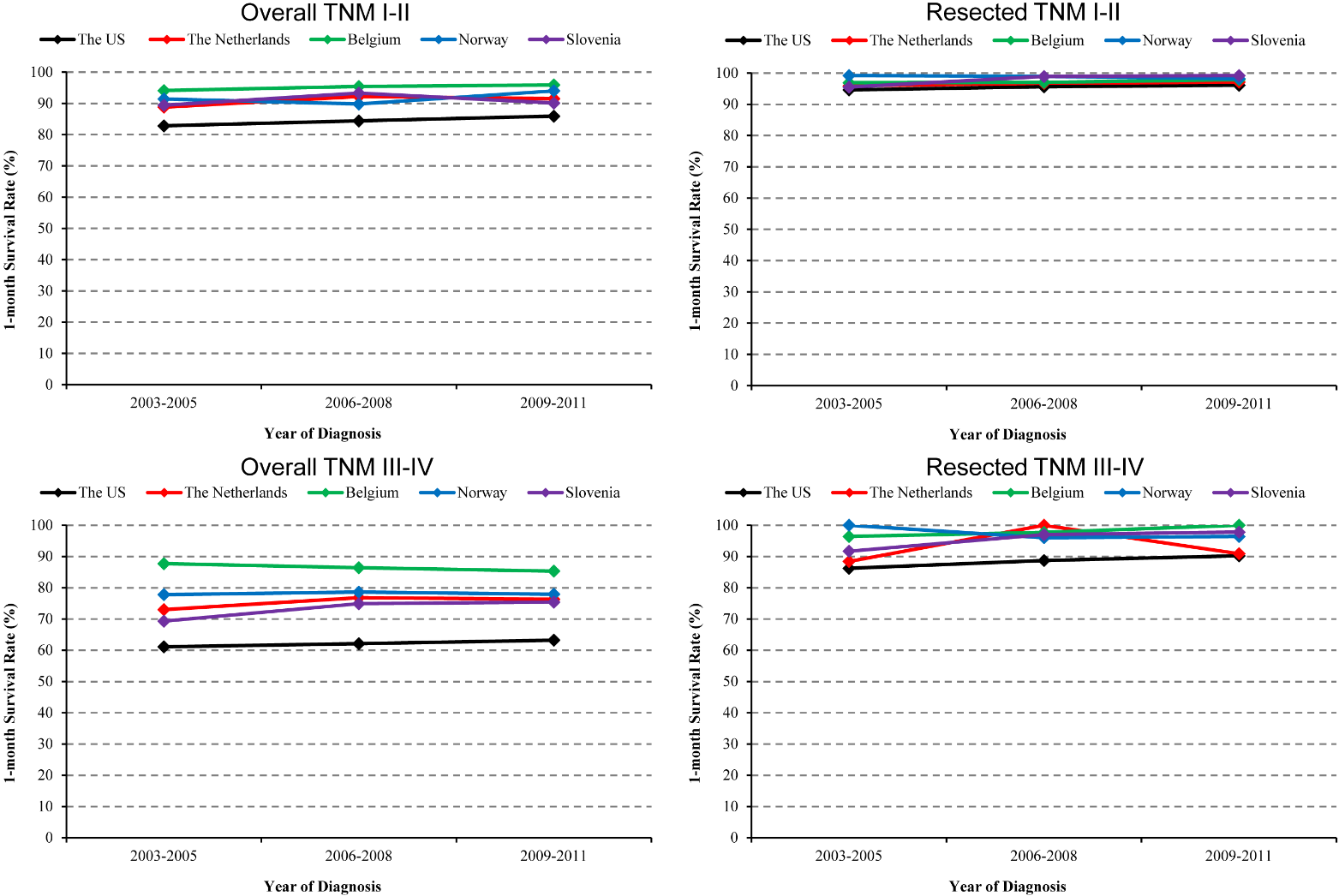


**Figure S3. Changes in 1-month survival over calendar periods among overall and resected patients with stages I-II and III-IV pancreatic cancers**


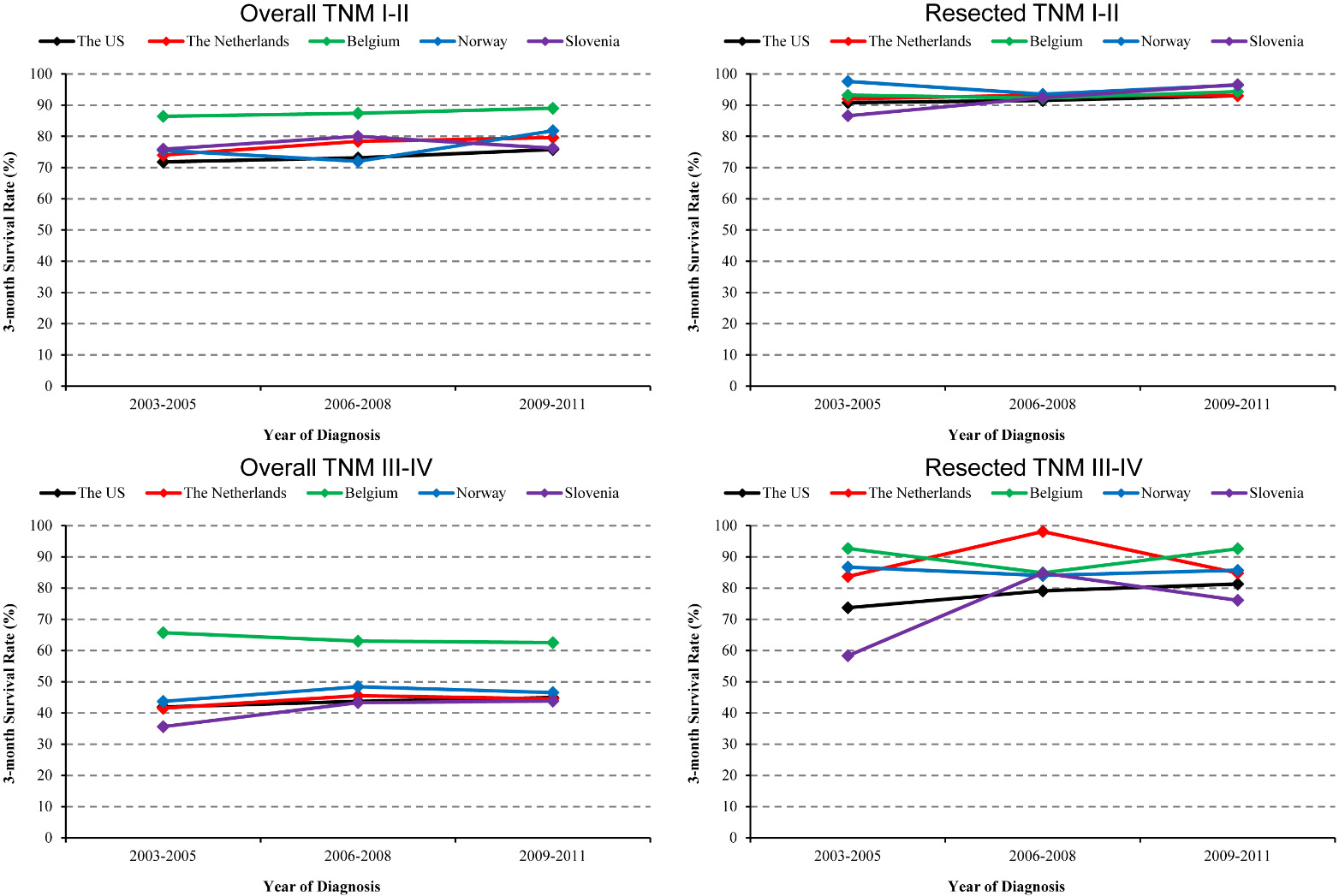


**Figure S4. Changes in 3-month survival over calendar periods among overall and resected patients with stages I-II and III-IV pancreatic cancers**


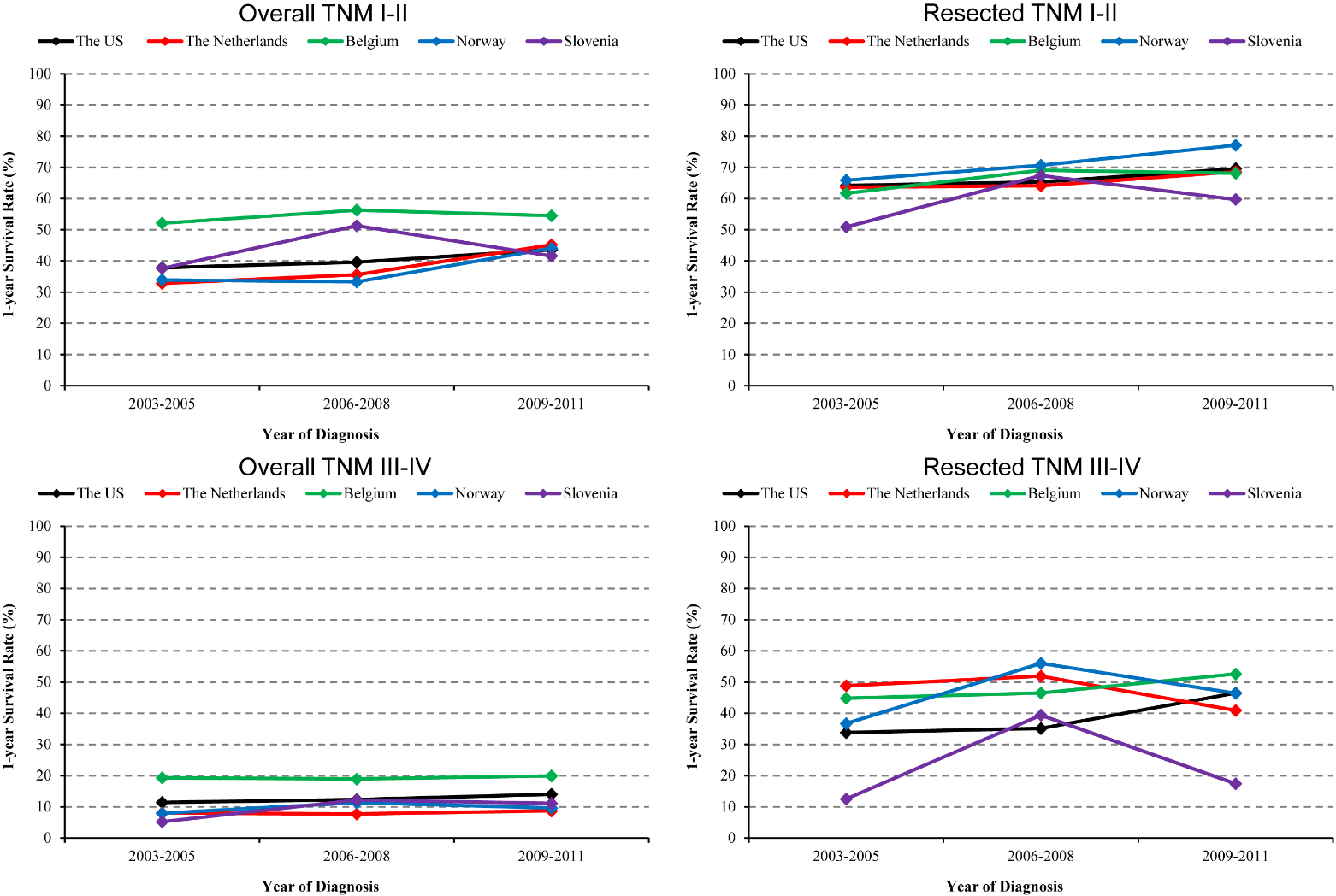


**Figure S5. Changes in 12-month survival over calendar periods among overall and resected patients with stages I-II and III-IV pancreatic cancers**


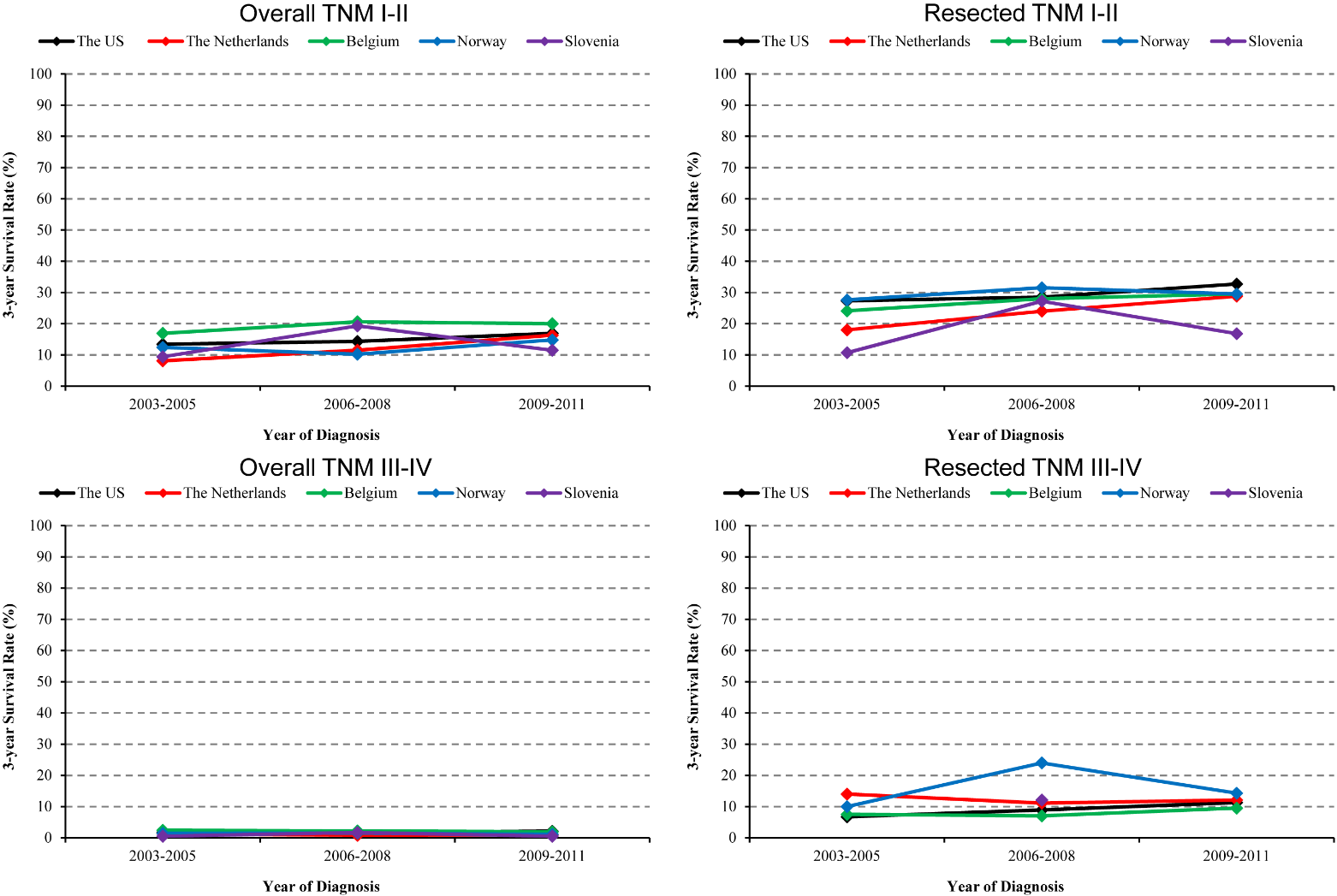


**Figure S6. Changes in 36-month survival over calendar periods among overall and resected patients with stages I-II and III-IV pancreatic cancers**


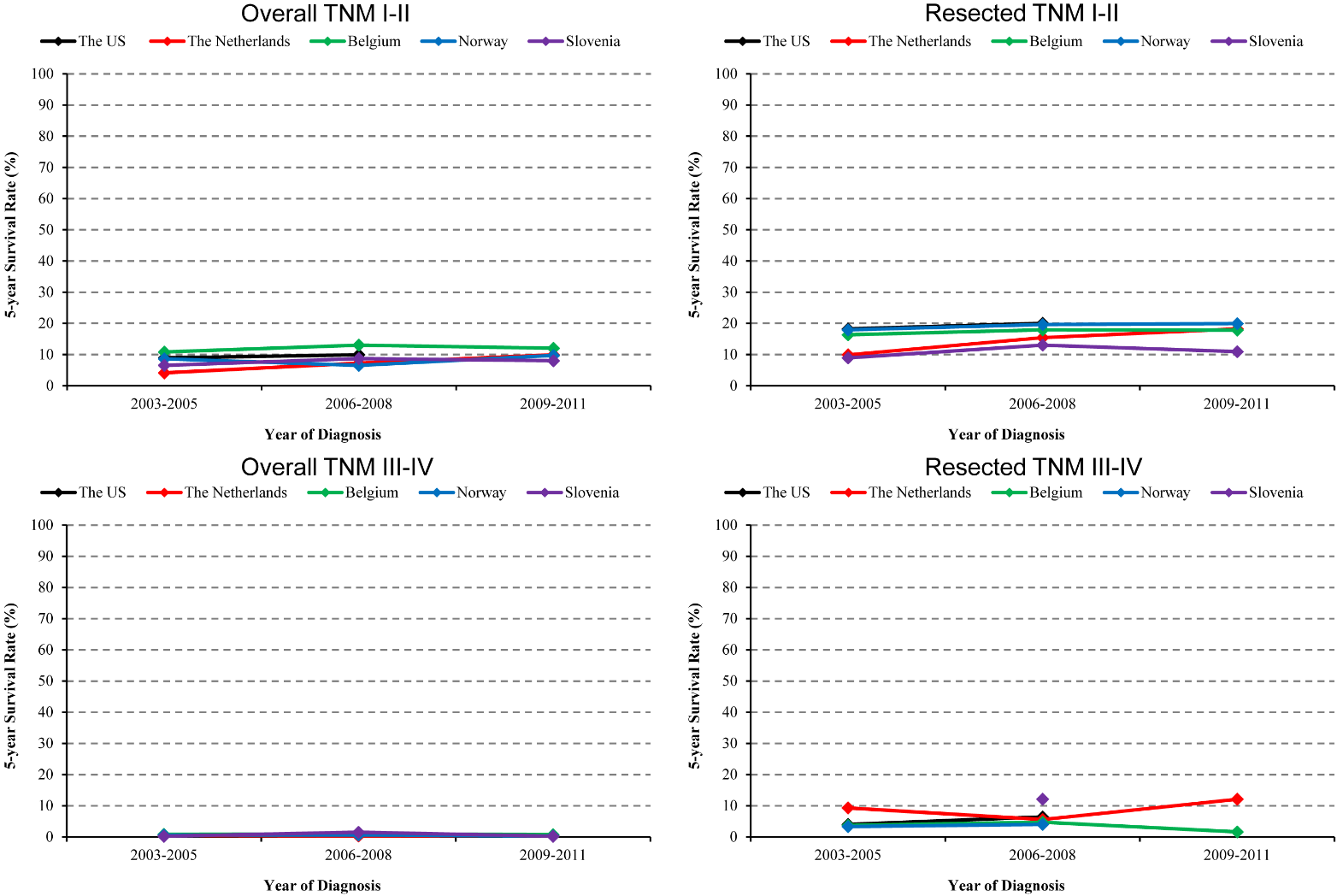


**Figure S7. Changes in 60-month survival over calendar periods among overall and resected patients with stages I-II and III-IV pancreatic cancers**
